# Reversed-Phase (RP) and Hydrophilic Interaction (HILIC) Separation Mechanisms for the Assay of Nicotine and E-Cigarette Liquids

**DOI:** 10.3390/molecules30163443

**Published:** 2025-08-21

**Authors:** Răzvan Moisi, Mircea-Alexandru Comănescu, Andrei-Valentin Medvedovici

**Affiliations:** Department of Analytical & Physical Chemistry, Faculty of Chemistry, University of Bucharest, Panduri Ave. #90-92, 050663 Bucharest, Romania

**Keywords:** chaotropic agents, ion pairing, reversed phase, HILIC

## Abstract

Nicotine is a highly used addictive substance that has recently also become available through electronic cigarettes. Here we present a study of nicotine from e-cigarette liquids through reversed-phase (RP) and hydrophilic interaction (HILIC) liquid chromatography. Multiple aqueous mobile-phase additives are considered for the RP mechanism, focusing on chaotropic agents, mobile-phase concentrations and mixing ratios, and column temperature. Sample preparation was conducted by toluene liquid–liquid extraction of e-cigarette liquids diluted with aqueous 25 mM NaHCO_3_/Na_2_CO_3_. Optimal RP results for retention and peak symmetry were obtained using aqueous 0.1% formic acid and 20 mM ammonium hexafluorophosphate with 0.1% formic acid in acetonitrile, using a gradient profile with a C18 column, exploited at 40 °C and a 1.5 mL/min flow rate. A dilute-and-shoot alternative with automated flow reversal after isocratic elution is presented. For HILIC, aqueous 100 mM ammonium formate and 0.1% formic acid in acetonitrile were used as mobile-phase components, using a gradient profile, on a Thermo Scientific™ Acclaim™ Mixed-Mode HILIC-1 column, operated at 25 °C with a 1 mL/min flow rate. UV detection was at 260 nm. Absolute limits of quantitation in the 1 μg/mL range were obtained for all tested alternatives, with 1 μL injection volumes.

## 1. Introduction

Nicotine is a naturally produced alkaloid found in various plants within the nightshade family [1], with tobacco plants generally producing the highest concentrations. Being an acetylcholine agonist, nicotine stimulates multiple bodily responses by binding to nicotinic acetylcholine receptors within the central and peripheral nervous systems, with one effect being the release of dopamine, which inadvertently plays a central role in the strong addictive effect of nicotine in humans [2,3,4,5,6,7,8]. While nicotine has been studied as an aid to memory and learning [9] for its stimulant effect, it is worth noting that its disadvantages likely outweigh the benefits, as nicotine use and exposure have also been linked to various cancers and cardiovascular illnesses [10]. However, nicotine has not yet been classified as a carcinogen.

Nicotine is still primarily consumed through tobacco products, such as smoking cigarettes, and snuffing or chewing tobacco. Within the last two decades, nicotine has also become available in the form of electronic cigarettes (e-cigarettes) [11]. Due to e-cigarettes not burning solid material but rather vaporizing nicotine-containing liquids (e-liquids), their use has been believed to be safer for both users and second-hand exposure [11,12], although a review of the literature [12] revealed a two-prong issue that e-cigarettes pose to public health. Firstly, while the hope is that e-cigarettes can lead to smoking cessation altogether, it is more indicative that their attraction to younger generations is increasing nicotine use. Secondly, while it is possible that less carcinogens are inhaled through vaporized e-liquids compared to cigarettes, there is an increase in cardiovascular and lung diseases, predominantly linked to the particulate created within the e-liquid vapor. Various studies have found that besides nicotine, other toxic organic compounds that can be found in the vapor produced by e-cigarettes are cancer-linked tobacco-specific nitrosamines (TSNAs) [13], formaldehyde and acetaldehyde likely formed by the degradation the e-liquid matrix (ethylene glycol, propylene glycol, glycerin) [14], toluene, ethylbenzene, xylene, acetone, acrolein, glyoxal [15], diacetyl and 2,3-pentanedione [16]. Due to the coil makeup in e-cigarettes, both e-liquids [17] and vapors [18] have also been found to contain various concentrations of heavy metals, which have been demonstrated to accumulate in the brains of mice exposed to e-cigarette vapors over a certain period [19].

Given the complexity of typical matrices that nicotine can be found in, such as natural products (loose-leaf tobacco) or synthetic mixtures (e-liquids), separation methods are typically required. Gas chromatography (GC) [20,21] has successfully been employed for the analysis of nicotine in solvent-extracted tobacco [20] or from e-liquids [21]. While this can be an alternative method of separation and analysis, liquid chromatography (LC) is also routinely employed for the investigation of nicotine from tobacco products. The choice of separation mechanism is ultimately dictated by the available laboratory equipment. Of course, given the fact that samples are no longer vaporized for liquid chromatography, one issue is posed by the viscosity of the e-liquids caused by polyethylene glycol and glycerin. Samples need to be diluted [22] and potentially extracted [23] prior to instrumental analyses. Due to its dibasic character (pKa1 = 3.41 and pKa2 = 8.10, log P = 1.17) [23,24], nicotine’s analysis in reversed phase [25] or, otherwise, its extraction from a more complex matrix must account for an increase in pH to draw it to a neutral form. This of course could cause issues with typical reversed-phase column longevities, as their typical pH range would be lower than the pyrrolidine nitrogen’s pKa, but applications of LC with mobile phases having a pH ranging between 7 and 9 have been successful in the separation of nicotine and some impurities in e-liquids on octadecyl-functionalized silicagel (C18, ODS) columns [22,25,26,27]. Two alternatives can take advantage of nicotine’s protonated form at low pH: the use of ion-pairing agents and separation in reversed phase [28,29], or simply the use of hydrophilic interaction liquid chromatography (HILIC) [30,31]. For ion-pairing (chaotropic) agents, tetrafluoroborate or hexafluorophosphate salts have been successfully used for separating and analyzing nicotine and its metabolites in human plasma, a fairly complex matrix, while HILIC has been proven useful in analyzing nicotine, metabolites and other related alkaloids in either neat [30], tobacco [31] or biological samples [32], with the latter demonstrating the need for extraction as well. Another possibility for enhancing retention in liquid chromatography is to take advantage of nicotine’s structure and pair it with a stationary phase such as phenyl-hexyl, which leads to π-π interactions for retention [33].

The amount of published information about LC separations of nicotine (and related compounds), analyzed in different matrices, is vast. From the selection presented in Supplementary Materials Table S1, one can conclude the following: **(a)** both RP and HILIC mechanisms are equally used in the separation of nicotine; **(b)** matrix complexity and reduced limit-of-quantitation (LOQ) values favor the use of high-resolution mass spectrometry (HRMS) or tandem mass spectrometry (MS-MS) detection; **(c)** the selectivity of MS or MS/MS detection systems is tunable, making the intrinsic selectivity of the chromatographic approaches less important (inadequate resolution between compounds is not a major concern even in quantitative approaches); **(d)** the selectivity induced by MS or MS/MS detection systems also makes less adequate peak symmetries tolerable, as peak purity can be easily checked; **(e)** the use of non- or less-volatile additives in the mobile phases and their increased concentration levels induce serious problems for coupling MS or MS/MS detection systems to LC separations; **(f)** trials using chaotropic agents and/or ionic liquids in the mobile-phase composition exist in the recent literature, but until now, no systematic approaches in correlating the nature and concentration of these additives had been found; **(g)** in trying to increase retention, some authors preferred replacing acetonitrile with methanol when using the RP mechanism; **(h)** in the HILIC mode, generally isocratic approaches were preferred, while the additives for the aqueous component of the mobile phase were chosen among ammonium formate and ammonium acetate buffers, with concentrations varying between 5 and 100 mM and pH values placed in the interval 2.3 to 7.0.; **(i)** in the HILIC mode, ambient column temperatures were usually preferred; **(j)** liquid–liquid extraction of nicotine from more or less complicated matrices is frequently used, with the extracting solvent being eliminated before injection; **(k)** dilute-and-shoot approaches appear in the literature, without a deeper evaluation of the column life span.

We proposed the comparison of the reversed-phase mechanism and HILIC, both studying qualitative aspects of the separation and demonstrating the quantitative applicability of the analysis of nicotine from e-cigarette samples. The novelty of this study consists firstly in the systematic approach to the role and effects of the additives used in the RP mode to separate nicotine. Different acidic additives, chaotropic in nature or not, taken alone or as mixtures were investigated for their role in increasing retention and optimizing peak shape and symmetry. Effects appearing on injection even when the volume was low (1 μL) or on volume increases were observed and discussed. For the dilute-and-shoot approach, an automated column wash process was proposed, based on flow reversal. The data presented supports that RP and HILIC modes behave similarly in terms of retention, selectivity and peak shapes. The RP mode allows higher flexibility in choosing the right elution conditions, according to the aim of each specific application. Direct injection of low volumes of samples diluted in solvents that are not miscible with the mobile phase in the RP mode was demonstrated, allowing a more straightforward way of designing the liquid–liquid extraction process used for nicotine isolation.

## 2. Results

### 2.1. RP Approach

The focus was to explore RP conditions ensuring a consistent retention and fair peak symmetry for nicotine, without considering classic ion-pairing agents (i.e., sodium alkyl sulfonates). The use of counterions with chaotropic properties was first considered. In Table 1 are listed the results (in terms of retention factors, symmetry factors, USP tailing factors and chromatographic efficiency) obtained in eluting nicotine from the ODS column through using different additives in the mobile phase and different organic modifiers. Phosphoric, perchloric and formic acid taken alone or in combination with potassium or ammonium hexafluorophosphate, or ammonium tetrafluoroborate, or in the presence of an ionic liquid (1-methyl-1-butylpyrrolidinium hexafluorophosphate—BMP) were considered. Elutions occurred in isocratic conditions, with a 95/5 (*v*/*v*) ratio between the aqueous and organic components of the mobile phase. The organic modifier always contained 0.1% of the acid used in the aqueous component. In Supplementary Materials Figure S1, the resulting overlaid chromatograms are also illustrated.

The best results in terms of retention, peak symmetry and efficiency were obtained when using aqueous 0.1% HCOOH + 20 mM NH_4_PF_6_/acetonitrile 0.1% HCOOH 95/5 (*v*/*v*) as a mobile phase. One can observe that the simple acidic addition to the mobile phase (0.1% H_3_PO_4_, HClO_4_ and HCOOH) led to an elution close to the void time (t_0_ = 1.072 min) of the chromatographic column. The use of the BF_4_^−^ anion produced less retention and a fronting effect on the nicotine peak, while the use of the ionic liquid led to the best peak symmetry, but produced a reduction in the relative retention.

It is interesting to note the effect observed with the addition of 0.1% HClO_4_ in the mobile phase, wherein there is a second peak with a reduced intensity eluting at a higher retention time and having the same UV spectrum as the main peak. Although the injection volume is low enough to exclude a sample solvent focusing effect, some additional attention should be paid to this. Supplementary Materials Figure S2 illustrates the resulting overlaid chromatograms after the injection of 1 μL volumes of 1000 μg/mL nicotine dissolved in methanol, acetonitrile and the mobile phase (aqueous 0.1% HClO_4_/acetonitrile 0.1% HClO_4_ 95/5 *v*/*v*). UV spectra taken at the times illustrated in the figure are also shown. As illustrated in Supplementary Materials Figure S3, this behavior is not observable when using 0.1% HCOOH addition in the mobile phase.

**Figure 1:**
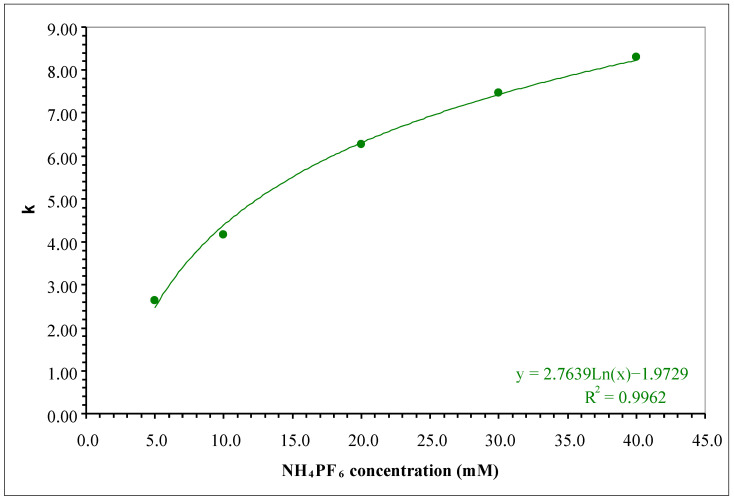
Variation in the retention factor characterizing the nicotine peak when varying the NH_4_PF_6_ concentration in the aqueous component of the mobile phase (isocratic elution conditions, aqueous/organic components 95/5 *v*/*v* with 0.1% HCOOH addition, 1 mL/min flow rate, 25 °C, 260 nm detection wavelength, 1 μL volume injected, nicotine concentration 1 mg/mL, sample solvent is methanol).

If the elution conditions with formic acid/ammonium hexafluorophosphate in the mobile phase produced superior results in relative retention and peak symmetry, the next step was to evaluate the concentration of the salt in the aqueous component. Figure 1 illustrates the variation in the retention factor characterizing the nicotine peak when using different concentrations of the ammonium hexafluorophosphate salt in the aqueous component of the mobile phase (variation in the 5 to 40 mM interval). The overlaid chromatograms, where the peak shape can be observed, are illustrated in Supplementary Materials Figure S4.

Retention increases logarithmically with the increase in the chaotropic salt concentration in the aqueous component of the mobile phase. However, for 5 mM NH_4_PF_6_, a strong dependence with respect to the injected volume and the sample solvent was observable. For repeated 1 μL volume injections of a nicotine solution of 1000 µg/mL in methanol, peak shape reproducibility was poor (see Supplementary Materials Figure S5). Reducing the injected volume to 0.5 μL avoided nicotine peak distortion, and the irreproducibility of the shapes disappeared. It is necessary to have a better insight into the effects appearing on injection in such elution conditions (reduced concentration of the chaotropic salt in the aqueous component of the mobile phase). Trials are illustrated in Supplementary Materials Figures S6 and S7.

**Figure 2:**
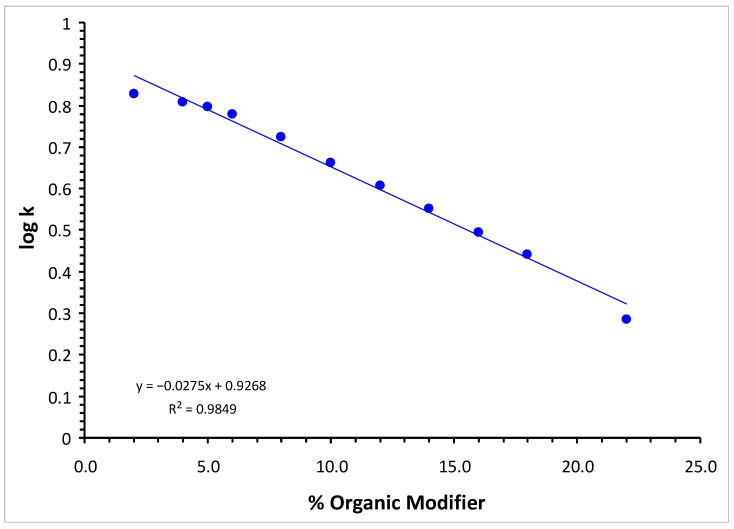
Plot of the logarithm of the retention factor characterizing the nicotine peak and the content of the organic modifier in the mobile phase (0.1% HCOOH), when using 20 mM NH_4_PF_6_ as chaotropic salt in the aqueous component only.

Modification of the retention of nicotine at different ratios between the aqueous and the organic components of the mobile phase, with 20 mM NH_4_PF_6_ and a 0.1% HCOOH aqueous phase, is illustrated in Figure 2. The overlaid chromatograms supporting the information in Figure 2 are illustrated in Supplementary Materials Figure S8. A linear variation is observable, as expected. The small deviation from linearity (correlation coefficient of 0.9924) may be explained by the overall variation in the chaotropic salt in the mobile phase according to the mixing ratio between components, if NH_4_PF_6_ was added (20 mM) only in the aqueous component.

**Figure 3:**
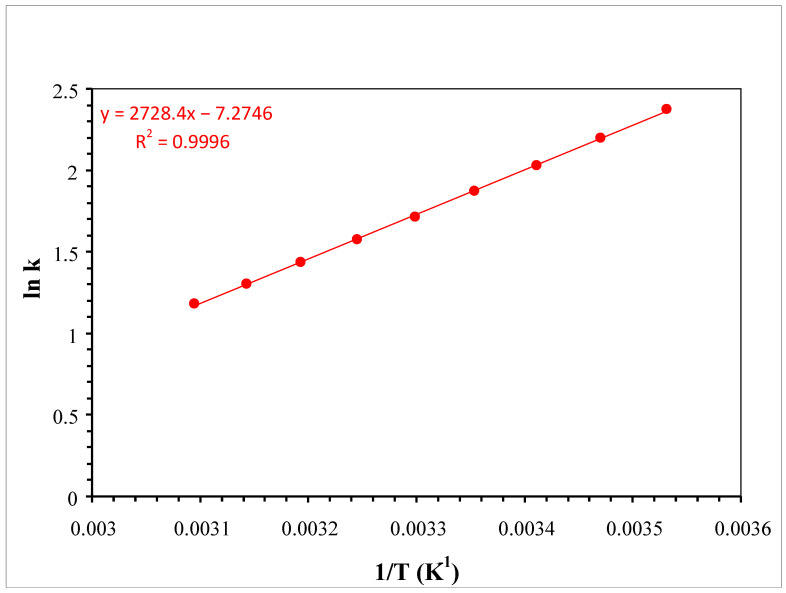
Resulting Van’t Hoff plot for nicotine under isocratic elution conditions (aqueous/organic: 95/5 *v*/*v*) when using 0.1% HCOOH in the mobile phase and 20 mM NH_4_PF_6_ as a chaotropic agent in the aqueous component of the mobile phase.

The influence of temperature on the elution of nicotine in RP conditions using chaotropic agent addition in the mobile phase was also studied. The Van’t Hoff plot resulting from the study is illustrated in Figure 3, with the overlaid chromatograms being illustrated in Supplementary Materials Figure S9. From the regression slope, one can calculate the enthalpy associated with the distribution of the analyte between phases ∆H^0^ as being −22.683 KJ K^−1^mol^−1^. If we approximate the volume of the stationary phase as being 0.2 mL and the volume of the mobile phase as the void time multiplied by the flow rate (1.072 mL), the variation in entropy ∆S^0^ associated with the process should be related to the intercept of the regression, obtaining a value of −46.52 JK^−1^mol^−1^.

It was also deemed important to check the retention behavior on other SunFire C18 (150 mm L × 4.6 mm i.d. × 3.5 µm d.p.) columns belonging to different stationary-phase production batches. These results are illustrated in Supplementary Materials Figure S10. One can observe from Figure S10 that parameters relating to retention, peak symmetry and chromatographic efficiency are characterized by relative standard deviations (RSD%) ranging from 3.1 to 6.7%. Such results can be considered fitting, taking into account that none of the tested columns were new at the moment of their use, each having been used for hundreds of varied sample injections before the study.

**Figure 4:**
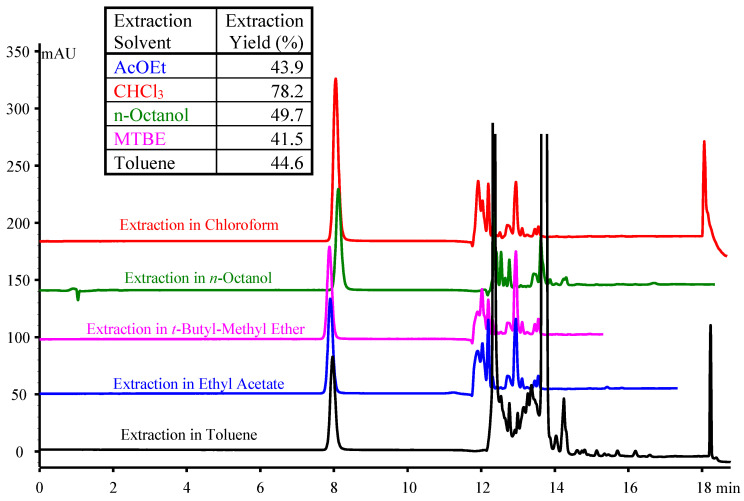
Chromatograms of the extracts made in toluene, ethyl acetate, t-butyl-methyl ether, n-octanol and chloroform of the Lemon Berry sample (20 mg/mL nicotine) (LB). Sample preparation protocol: dilution 1/100 (*v*/*v*) of the Lemon Berry sample in aqueous 25 mM Na_2_CO_3_ and NaHCO_3_, followed by extraction in the organic solvent (aqueous/organic ratio 4/1 *v*/*v*). Injection: 1 µL from each organic layer. Mobile phase: aq. 0.1% HCOOH + 20 mM NH_4_PF_6_/ACN 0.1% HCOOH. Elution profile: 5% organic (9 min) up to 100% organic at min 11 for at least 6 min.

The influence of the extraction solvent was also considered. The main aim was to observe the influence of the extraction solvent on the chromatographic peak profiles, rather than focusing on evaluation of the extraction yields. At least theoretically, one-microliter injection volumes should not have major impacts on peak profiles, unless the solvents are immiscible with the mobile phase. The tested extraction solvents were (a) ethyl acetate (AcOEt); (b) chloroform; (c) *n*-octanol; (d) methyl tert-butyl ether (MTBE); (e) toluene, with 1 µL of the extracts injected, using the Lemon Berry 20 mg/mL sample (LB). The elution profile was modified after 9 min of an isocratic run at 5% organic modifier in the mobile phase, up to 100% organic modifier in 2 min, followed by a column wash for at least 8 min. Results are illustrated in Figure 4.

**Figure 5:**
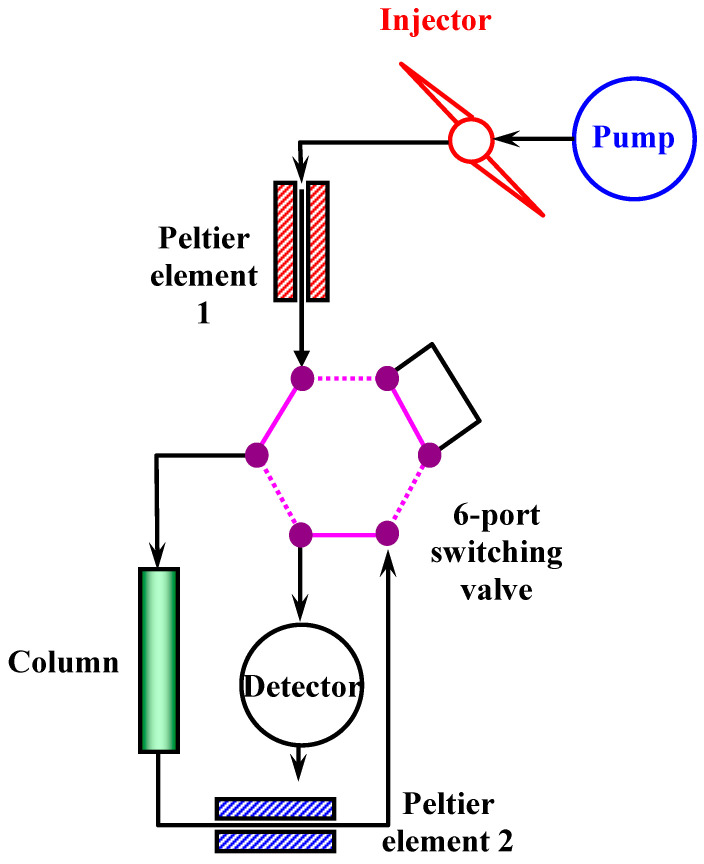
Design using the column switching valve to reverse the flow in the column immediately after elution of a peak of interest, allowing a fast elimination of the sample matrix and reducing its accumulation in the stationary phase over multiple consecutive sample injections.

A final test on RP was performed by directly injecting 1:10-diluted LB and BI-0 matrices. After 12 min, the flow was reversed using the six-port column switching valve, with the mobile-phase composition being set to 100% ACN to help eliminate matrix components that would accumulate at the beginning of the column. Both elution stages were monitored via the DAD. The connections to the valve are illustrated in Figure 5. We did not observe column issues after the ten injections, as can be observed from the nicotine retention (0.49% RSD) (see Supplementary Materials Figure S11A). The BI-0 e-liquid chromatograms revealed a peak elution at 9.954 min (see Supplementary Materials Figure S11B), whose spectra is attributable, based on the literature [34], to maltol. Maltol is declared by the producers to be in the BI-0 e-liquid but is not listed in the LB composition.

Given the complexity of the e-liquid matrix, it is not recommended to dilute and shoot without column reverse washing, as it may cause column clogging, modification of column behavior (e.g., increasing back pressure, peak shape deterioration, abnormal retention time shifts, baseline shifting, etc.) and shortening of the column lifetime.

### 2.2. HILIC Approach

The HILIC approach for nicotine separation was achieved on a stationary phase consisting of a chemically modified silicagel with dodecyl moieties (C12). The last two carbon atoms from the linear chain are substituted with two (vicinal) hydroxyl groups. The material behaves bimodally, as a RP mechanism with aqueous rich mobile phases and as a HILIC mechanism with organic rich mobile phases.

Isocratic approaches to eluting nicotine under HILIC conditions produce enough retention but inappropriate peak profiles (symmetries). Supplementary Materials Figure S12 illustrates elution of nicotine with 95% organic modifier containing mobile phase at 30 °C.

In such conditions, a gradient elution profile should be used as a tool to improve peak symmetry. Two gradient elution profiles have been considered. The first profile (A), running at 25 °C, starts at 95% organic modifier (kept constant for 1.5 min) followed by a linear decrease of 10%/min to 60% organic modifier (kept constant again for 2 min). The second profile (B), running at 50 °C, starts at 98% organic modifier (kept constant for 2 min) followed by a linear decrease of 7%/min to 63% organic modifier (kept constant again for 5 min). The resulting overlaid chromatograms are illustrated in Supplementary Materials Figure S13. One can observe that both gradient profiles behave somewhat similarly in terms of retention and peak symmetry. Elution at 50 °C certainly enhances the apparent efficiency. As one can observe from Supplementary Materials Figure S14, illustrating part of the peak tool report (from ChemStation’s “macro tools.mac”), peak symmetry values are quite similar, regardless of the gradient profile. Considering that a 50 °C exploiting temperature regime would considerably reduce the column lifetime, gradient profile A was chosen for further continuing investigations.

The influence of the column equilibration time on nicotine elution under HILIC conditions with the gradient A profile was also investigated, in an interval ranging from 1 min to 20 min. Results are illustrated in Supplementary Materials Figure S15. Although after a 1 min equilibration period the initial pressure drop on the column is not perfectly restored, one can observe that an excellent precision is obtained for the absolute retention time (min), apparent retention factor (void time is considered as retention time of toluene), peak area (mAU*s), peak symmetry at 10% of the height and USP tailing factor. With respect to gradient profile B, equilibration time variations generate reduced retention and a significant reduction in the apparent peak efficiency, as illustrated in Supplementary Materials Figure S16.

Another aspect investigated was the injected volume, once the sample was extracted in toluene. Injection volumes ranging from 1 to 10 µL were utilized. As illustrated in Supplementary Materials Figure S17, both absolute retention and peak symmetries exhibit similar values up to a 5 µL injection volume. After 5 µL, some loss of peak symmetry and apparent efficiency may be observed, however, without serious deterioration of the chromatogram. Finally, the repeat injections of the 1000 ppm nicotine indicated highly reproducible peak characteristics. These results are illustrated in Supplementary Materials Figure S18.

### 2.3. Quantitative Studies

Supplementary Materials Table S2 presents the dilution and extraction results following the use of the LB e-liquid sample. As can be seen, peak area comparisons show that the neat carbonate buffer had higher extraction efficiencies at each sample/toluene volume ratio, except when compared with the salt-saturated carbonate buffer at a 2/1 extraction ratio. Given the overall trend of the RSDs exhibited by the salt extractions, it may be that the salt presence induces poorly reproducible extractions. While the 4/1 neat carbonate buffer extraction had a high RSD, its lowest recorded area value is still comparable with the salt-saturated counterpart’s average. As such, a 4:1 (*v*/*v*) sample/toluene extraction protocol with the carbonate buffer as a sample diluent was considered a good option for further investigations.

In order to assess the accuracy of the extraction process of nicotine from the e-cigarette liquids over a large interval of concentrations, it was studied for neat solutions in methanol for both RP and HILIC alternatives, as well as through spiking the 0 mg/mL declared e-cigarette fluid (BI-0) after dilution by a factor of 1/100 (*v*/*v*) according to the experimental design described under Materials and Methods; the stock spike of a 1000 μg/mL level was diluted with the same 1/100-diluted matrix in order to obtain solutions with concentrations in the 1–1000 μg/mL range. These solutions were extracted with toluene in a volumetric ratio of 4/1 (*v*/*v*). The resulting toluene extracts were injected under RP and HILIC conditions, nicotine peaks were integrated, and the data was processed using both a simple linear regression and linearly weighted regression (by 1/x^2^). The manufacturer-declared unspiked blank e-liquid was also extracted. During analysis, we observed that the unspiked matrix had a small nicotine signal (equivalent to a signal of about 0.74 µg/mL when using the neat RP weighted regression). As such, the data used for the extraction calibrations was corrected by blank signal subtraction. The resulting regression data are listed in Table 2. Formulas used for the calculations [35] are listed in Supplementary Materials Table S3. The calibrations are presented in Supplementary Materials Figures S19−S22.

Table 3 presents the results and calculated extracted concentrations (ppm) from the 20 mg/mL tested e-liquids, based on the linear weighted regressions developed from the spiked matrices. Given that the samples were also 100-fold diluted, the expected concentration should be 200 ppm, showing that both developed methods had comparable biases (within ±10%) for four of the five samples.

## 3. Discussion

From our mobile-phase studies of the reversed-phase mechanism, it is obvious that the best results in terms of retention, peak symmetry and efficiency were obtained when using aqueous 0.1% HCOOH + 20 mM NH_4_PF_6_/acetonitrile 0.1% HCOOH. When considering the potential ion-pairing mechanism, other counterions (BF_4_^−^, ClO_4_^−^) were not equivalent, nor was the use of ionic liquid as an aid in retention. On closer investigation of addition of 0.1% HClO_4_ in the mobile phase, two peaks were seen to develop, and the effect was not attributed to solvent focusing. It might be possible that the voluminous perchlorate anion pairing of the two protonated nitrogen atoms from the nicotine molecule results in restriction of the free rotation against the sigma bond existing between the pyridine and N-methyl-substituted pyrrolidine heterocycles, leading to stabilization of rotamers (Supplementary Materials Figure S23).

The results illustrated in Supplementary Materials Figures S5−S7 clearly indicate the formation of nicotine conformers, more or less stabilized in the elution conditions characterized by a low concentration of the chaotropic counterion PF_6_^−^, according to the sample solvent and injection volume being used. The “classic” recommendation to use the mobile phase as sample solvent for generating reproducible results on increasing the injection volume is also valid in this situation. This shows that solvent focusing effects can be observed even in the case of reduced injected volumes, in the case of using chaotropic additives in the mobile phase.

The Van’t Hoff data regarding the influence of the temperature on the elution of nicotine in RP conditions through using chaotropic agent addition in the mobile phase indicates that the distribution of nicotine between the stationary and mobile phases is enthalpy-controlled and based on adsorption processes.

One can observe that the solvent used in the extraction process does not have an influence on the peak profile and symmetry, while the influence on retention is minor. A rough calculation of the extraction yields shows that chloroform behaves better (around 78%), followed by octanol (around 50%), while the other three solvents produce extraction yields in the interval of 41 to 45%. However, taking into account that the chloroform layer is the bottom layer in the Eppendorf extraction vial, its retrieval is more cumbersome; conversely, octanol requires a longer period of acetonitrile column washing in order to evacuate the injection solvent plug from the column [36], and thus, attention was shifted toward toluene, whose elution from the column can be simply monitored at the recorded wavelength.

Given that HILIC applications require a uniform water layer on the stationary phase, the post-gradient equilibration time was studied. The findings indicate a better tolerability for equilibration time variations in gradient profile A compared to B; the first presents reproducible results in terms of retention and peak symmetry irrespective of the period of equilibration between runs.

Determination of the diluent choice compared neat carbonate and sodium hydroxide solutions, as well as their sodium chloride salt-saturated counterparts. The salt-saturated solutions were used in an attempt to see whether a salting-out effect could be forced in increasing the extraction efficiency. Compared to the other trials, the neat carbonate buffer was deemed a good choice of diluent. While sensitivity was not considered an issue given that e-cigarettes have high nicotine concentrations, the samples were nonetheless processed to also enhance sensitivity by choosing the 4/1 (*v*/*v*) sample-to-toluene volume ratio, thus theoretically having a 4x concentration factor.

Based on the observed calibration data, taking into account that the concentration interval is relatively large, the simple linear regression model fails to produce acceptable back-interpolated values (and consequently % biases), especially at the lowest concentration levels (due to the saturation of the response of the detector), despite having fairly good correlation coefficients (> 0.99); consequently, the computed LOQ values, regardless of the calculation method, are generally placed in the 25 to 100 μg/mL interval. When applying the weighted 1/x^2^ linear regression model, the back-interpolated values and the corresponding % biases fall in the +/−15% interval; LOQ values consequently have the tendency to be reduced and placed in the 1–5 μg/mL concentration interval. Further, when applying the weighted regression model (more precisely in the case of nicotine toluene extracts processed in both RP and HILIC separation mechanisms), due to a small intercept increase, applying the LOQ(3) formula (LOQ = (10 × s_A_ − A)/B) results in negative values (having no physical meaning).

The slopes of the weighted linear regressions obtained through injection of neat methanolic solutions are fairly similar (1.5206 in the RP mechanism and 1.3251 in the case of the HILIC separation mechanism), and the same situation can be observed for the slopes of the weighted linear regression resulting from injection of the nicotine toluene extracts under the RP and HILIC separation mechanisms (2.9429 for RP and 2.6878 for HILIC). The ratios between the slopes of the weighted linear regressions of the toluene extracts and those resulting from injection of neat methanolic solutions of nicotine are also in fair accordance (1.94 for RP and 1.85 for HILIC). Considering that the experimental procedure results in a concentrating factor of 4, comparing this value with the ratios of the slopes (extract vs. neat), one can conclude that the recovery of nicotine in the toluene extraction process is 48.4% (RP) or 46.3% (HILIC). These values are in fair accordance with the recovery of 44.6% determined in the preliminary experimental procedure illustrated in Figure 4. The data characterizing the linear regressions obtained for the extracts of nicotine in toluene indicates that the analytical process behaves similarly regardless of the initial concentration of the analyte in the initial matrix. The sensitivity was not considered a major concern in this application as the available e-cigarette liquids contain high concentrations (20 mg/mL) of nicotine. Nonetheless, the LOQs determined using the weighted approach are placed between 0.1 µg/mL (RP) and 1.0 µg/mL (HILIC). Given the dilution and concentration step, this would essentially mean that LOQs for neat samples would be around 2.5 µg/mL and 25 µg/mL, respectively. The sensitivity can theoretically be increased by another factor of 5 (0.5 µg/mL and 5 µg/mL) if the injection volume is increased from 1 µL to 5 µL. Although this study presents only a demonstration of quantitative applicability, not having gone through method validation, a comparison to FDA and WHO [37,38] procedures for nicotine analysis in tobacco products shows that the presented method falls within a ±15% bias when using the weighted linear regression, with R^2^ values higher than 0.99, and working ranges and LOQs lower than those proposed (1–30 mg/mL with 0.2 mg/g LOQ [38]); note, however, that these values are determined for GC-FID analyses.

An evaluation of the e-liquid components revealed that, while each e-liquid has two to four unique ingredients, when considering the common ingredients, samples LB and CT are close, with LB lacking only vanillin and piperonal, which in our opinion should not affect the extraction efficiency. This might be an indication that the sample may have a less than 20 mg/mL nicotine content.

A legitimate question can be addressed after optimizing the whole extraction process of nicotine from e-cigarette liquids and its assay (for quality control purposes): why do we need extraction? The complexity of the nicotine-containing liquids for e-cigarettes is certainly a major problem relating not only to the intrinsic selectivity of the chromatographic process in itself but also to the major components, such as propylene glycol and glycerin, accumulating on the column after repeat injections, such as during automated quality control processes.

To verify whether an alternative assay of nicotine in e-cigarette liquids may be possible by simply injecting a diluted aliquot from the original sample without risking irreversible column damage by excessive loadings, we tested two samples, a 20 mg/mL (LB) and a 0 mg/mL (BI-0), by a tenfold dilution and direct injection. After 12 min, the column flow was reversed, and the solvent was switched to 100% organic. As observed in Supplementary Materials Figure S11, upon reversing the column flow, various non-identified peaks are detected, indicating accumulation of the sample matrix at the beginning of the column. This means that direct dilute-and-shoot repeat tests, without post-injection reversed-flow cleaning, will eventually lead to column issues and data irreproducibility. We demonstrated that the workaround for this is the use of the six-port column switching valve, programmed for automated reverse flow to facilitate automatic analysis and prolonging of the column lifetime and characteristics (Figure 5).

## 4. Materials and Methods

### 4.1. Chemicals and Reagents

The following reagents were used in this study: ammonium hexafluorophosphate (≥98.0%, Fluka Analytical, Buchs, Switzerland), ammonium formate (puriss p.a., Fluka Analytical), methanol (gradient grade, Sigma-Aldrich, St Louis, Missouri, USA), toluene (ACS grade, Fluka Analyitical), acetonitrile (Chromasolv, ≥99.9%, Sigma-Aldrich), formic acid (extra pure, Merck, Merck, Darmstadt, Germany), sodium hydroxide (puriss p.a., Sigma-Aldrich), sodium carbonate (Pro Analysis, Merckx), sodium bicarbonate (Reactivul București), nicotine (for biochemistry, ≥99.9%, Roth GmbH, Heilbronn, Pleidelsheim, Germany), chloroform (UVasol, Merck), octanol (Emplura, Merck), ethyl acetate (LiChrosolv, Merck), methyl tert-butyl ether (LiChrosolv, Merck), perchloric acid (puriss. p.a., Sigma-Aldrich), phosphoric acid (85–90%, for HPLC, Fluka Analytical), potassium hexafluorophosphate (≥98.0%, Fluka Analytical), ammonium tetrafluoroborate (≥99.999%, Aldrich), 1-methyl-1-butylpyrrolidinium hexafluorophosphate (≥97.5%, Aldrich), potassium nitrate (p.a., PanReac AppliChem, Darmstadt, Germany) and sodium chloride (pure, Poch, Gliwice, Poland).

HPLC-grade ultrapure water (resistivity ≥ 18.2 MΩ × cm, TOC ≤ 5 ppb, microorganisms ≤ 0.001 CFU/mL) was obtained using a Nuzar-Q ultrapure water system (RephiLe Bioscience, Boston, MA, USA).

### 4.2. Equipment

Chromatographic separations and analysis were performed on an Agilent 1260 system (Santa Clara, CA, USA), equipped with a quaternary pump with a four-channel degasser (G1311B), an automated liquid sampler fitted with a 100 µL injection loop (G1329B), a column thermostat (G1316C) and a multi-channel diode array detector (G1365D). System control, data acquisition and data processing were performed through Agilent ChemStation LC3D (04.03(16)) software. To achieve the design illustrated in Figure 5, a high-pressure 6-port switching valve G1353-68750 controlled via Chemstation software was integrated in the column thermostat module. Detection was achieved through using the deuterium lamp spectral source and monitoring of the analytical wavelength of 260 ± 4 nm, with 560 ± 10 nm as a reference wavelength. Spectra were continuously acquired during chromatogram acquisition over the interval 210–500 nm, with a spectral window of 2 nm.

Vortex mixing operations were achieved on a Stuart SA8 (Sigma-Aldrich) vortex. Centrifugation operations used for phase separation were achieved through using a MiniSpin 5452 Eppendorf AG (Hamburg, Germany) centrifuge operated at 13,400 RPM (12,100× *g*) for a ten-minute centrifugation duration.

### 4.3. Chromatographic Conditions—Reversed Phase

Reversed-phase analyses were performed on a Waters SunFire C18 column (150 mm L × 4.6 mm i.d. × 3.5 µm d.p.) serial no. 01653815614-092. For confirmation of the reproducibility of the separation approach, two other Waters SunFire C18 columns (150 mm L × 4.6 mm i.d. × 3.5 µm d.p.) packed with different stationary-phase production batches, 013930085136-73 and 015233239140-86, were tested.

For qualitative approaches, isocratic elution with a 95/5 (*v*/*v*) ratio between the aqueous and organic components of the mobile phase, at 25 °C and a flow rate of 1 mL/min, was used. The aqueous phases consisted of phosphoric, perchloric and formic acid taken alone or in combination with potassium or ammonium hexafluorophosphate, or ammonium tetrafluoroborate, or in the presence of an ionic liquid (1-methyl-1-butylpyrrolidinium hexafluorophosphate—BMP). The organic modifier always contained 0.1% of the acid used in the aqueous component. An injection volume of 1 μL of a nicotine 1000 μg/mL solution in methanol was used. Additional tests explored the effects of the ammonium hexafluorophosphate concentration in the aqueous phase, the volumetric ratio of aqueous/organic mobile phase components and the column temperature, as well as behavior on three different columns of the same make.

During optimization of the sample extraction solvent, the mobile phase was composed of aqueous 0.1% formic acid, with 20 mM ammonium hexafluorophosphate (Solvent A) and 0.1% formic acid in acetonitrile (Solvent B). Elution was kept isocratic at 5% Solvent B for 9 min, followed by a gradient up to 100% Solvent B in two minutes, with composition maintained constant for another eight minutes. Column temperature was set at 25 °C. Injection volume was 1 μL.

For analysis of the extracted samples and calibrations, the same mobile-phase components as stated above were used (aqueous 0.1% formic acid and 20 mM ammonium hexafluorophosphate—Solvent A; and 0.1% formic acid in acetonitrile—Solvent B). The column was operated at 40 °C and a 1.5 mL/min flow rate. The gradient profile started with 10% organic modifier for 1.5 min, followed by a 15%/min increase to 62.5% organic modifier. A further gradient step up to 100% Solvent B in one minute followed, kept constant for three minutes. Column re-equilibration for at least four minutes was necessary before the subsequent injection. RP quantitative analyses used neat calibration samples prepared by serial dilutions starting from 1000 μg/mL nicotine in methanol (eight calibration levels ranging from 1 to 1000 μg/mL).

For the dilute-and-shoot approach, elution was made isocratic for 12 min at 5% Solvent A. When the valve switched at minute 12 for flow reversal, the composition of the mobile phase shifted instantaneously to 100% Solvent B. Column temperature was 25 °C, flow rate 1 mL/min and sample injected volume 1 μL.

### 4.4. Chromatographic Conditions—HILIC

For HILIC analyses, a Thermo Scientific™ Acclaim™ Mixed-Mode HILIC-1 (150 mm L × 3 mm i.d. × 3 µm d.p.) column was used, with a 1 mL/min flow. For gradient profile A, the column was thermostated at 25 °C, and the mobile phase consisted of aqueous 100 mM ammonium formate, pH-adjusted to 3.5 with formic acid (as aqueous component), and 0.1% formic acid in acetonitrile (as organic modifier). For gradient profile (B), the column was thermostated at 50 °C, and the aqueous component of the mobile phase was the same, while the organic modifier consisted of acetonitrile. Gradient profile A started at 95% organic modifier for 1.5 min, followed a 10%/minute decrease to 60% organic modifier, kept constant for 2 min, followed by column equilibration. Gradient profile B started with 2 min of 98% organic modifier, followed by a 7%/min decrease to 63% organic modifier, kept constant for 5 min, followed by column equilibration. HILIC analyses included comparisons between gradient profiles A and B, injection repeatability, column equilibration time effects between 1 and 20 min, and injection volume effects using 1, 2, 5, 7 and 10 µL injections, with 1000 ppm nicotine in toluene, and quantitative neat calibration samples of nicotine in methanol (eight calibration levels, ranging from 1.1 to 550 ppm).

### 4.5. Samples

Seven “Vuse GO” British American Tobacco e-cigarettes were also purchased, six with a 20 mg/mL declared nicotine concentration, and one zero-nicotine e-cigarette (0 mg/mL). The e-cigarettes had different aromas, with two being Lemon Berry (LB), and one each of Creamy Tobacco (CT), Blueberry Ice (BI), Berry Twist (BT) and Blue Raspberry (BR). The zero-nicotine e-cigarette’s flavor was Blueberry Ice (BI-0).

### 4.6. Sample Preparation

For the dilute-and-shoot scenario, the sample (Lemon Berry) or the blank (Blueberry Ice) was diluted 1:10 (1/2/7 *v*/*v*/*v* of sample/organic mobile phase component/aqueous mobile phase component) and directly injected onto the column after vortex mixing. Ten injections (five blanks and five samples) were performed as described earlier in Section 4.3.

**Figure 6:**
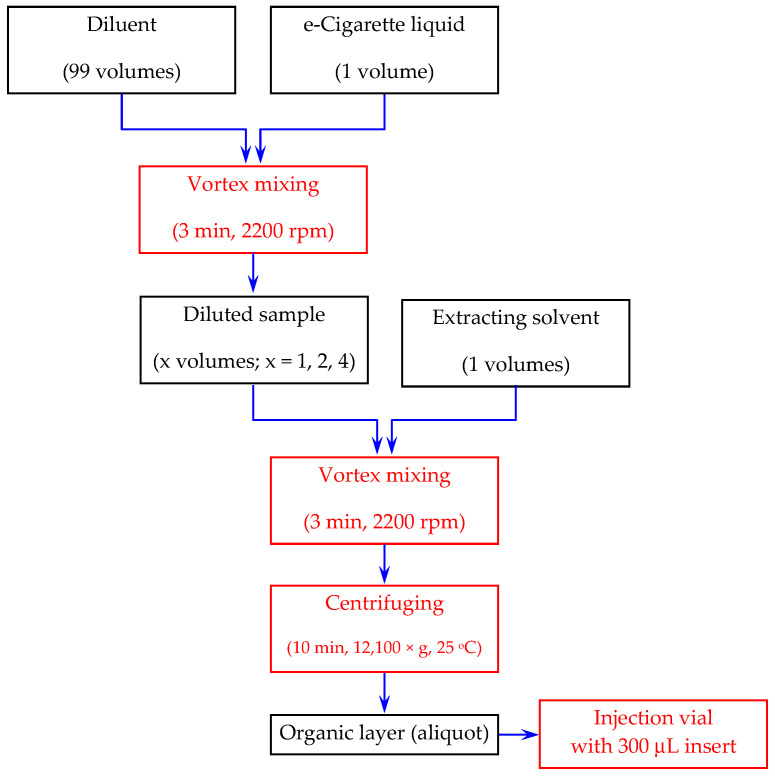
Operational flow chart describing extraction of nicotine from e-cigarette liquids.

For the liquid–liquid extraction scenario, the overall operation flow chart used for sample extraction is illustrated in Figure 6.

During optimization of the extraction process, the following parameters were considered: (i) the composition of the diluent; (ii) the nature of the extraction solvent; (iii) the volumetric ratio between the diluted sample and the extraction solvent.

Four diluents were compared: (a) aqueous 25 mM NaHCO_3_/Na_2_CO_3_; (b) aqueous 25 mM NaHCO_3_/Na_2_CO_3_ saturated with sodium chloride; (c) aqueous 0.1 N sodium hydroxide solution; (d) aqueous 0.1 N sodium hydroxide solution saturated with sodium chloride.

Five extracting agents were considered for preliminary evaluation: chloroform, n-octanol, t-butyl-methyl ether, ethyl acetate and toluene. Chloroform provided an extraction yield close to 80%, while all other solvents produced extraction yields ranging from 41.5% to 50%. In all cases, for the organic layer, although immiscible with the mobile phase, 1 μL of the extract was directly injected onto the chromatographic column without inducing any effects on nicotine’s retention and peak shape/symmetry. Despite the lower extraction yield obtained in toluene compared to chloroform, the first was preferred because of the following reasons: (i) the organic layer is on top, allowing easier removal of the aliquot; (ii) the lower extraction yield in toluene is acceptable, as the method’s sensitivity is not a major concern, with the concentration of nicotine in e-cigarette liquids being high; (iii) elution of toluene in both RP (at a much higher absolute retention time compared to nicotine) and HILIC (eluting at the column’s void time) separation modes can be easily observed at the monitored wavelength, allowing stricter control of the chromatographic run duration and/or optimization of the gradient profiles.

The volumetric ratios between toluene and the diluted sample were 1:1, 2:1 or 4:1 (*v*/*v*).

Calibration samples were produced by using the zero-nicotine e-cigarette Blueberry Ice (BI-0) liquid, 100-fold diluted with aqueous 25 mM NaHCO_3_/Na_2_CO_3_. The diluted blank sample was spiked with standard nicotine stock solution to produce a 1000 μg/mL sample. Serial dilutions were made to produce 500, 100, 50, 25, 10, 5 and 1 μg/mL solutions of nicotine in the diluted blank sample solution. These were then extracted with toluene in a 4:1 volume ratio (1 mL sample to 250 µL toluene) and analyzed via both RP and HILIC gradient methods.

The nicotine-containing e-liquid samples were similarly analyzed by 100-fold dilution and 4:1 (*v*/*v*) extraction using both RP and HILIC methods.

## 5. Conclusions

Nicotine has been used for human consumption through various means, from natural forms (tobacco leaves) to, now, more synthetic methods via vaporization of e-liquids, or use of nicotine patches.

The matrix in which nicotine is to be assayed will directly influence the sample preparation and chosen chromatographic method. When volatility may act as a discriminant factor against the matrix, GC will likely be the best chromatographic solution. Isolation of nicotine from complex matrices of natural origins (from botanical sources to biofluids and tissues) requires more elaborate sample preparation approaches, from liquid–liquid to solid-phase or solid–liquid extraction (LLE, SPE, SLE), including protein precipitation alternatives and possible process automation (when the sample number to be handled is high). In such instances, LC may act as a challenging alternative for the chromatographic stage. Isolation of nicotine from aqueous media in solvents that are not miscible with water will require alkalinization of the extracted phase, to inhibit protonation of the basic moieties in the analyte structure.

The choice of chromatographic detection is directly tied to the required sensitivity. While Flame Ionization Detectors (FIDs) or Nitrogen–Phosphorous Detectors (NPD) represent the basic choices for GC separations and UV is the alternative for the LC counterpart, when the required sensitivity goes down to the ng/mL–pg/mL level, (HR)MS and MS/MS are the only alternatives to be considered for both GC and LC approaches.

From the experiments described herein, we observed the following: (a) The RP separation mode is more flexible compared to the HILIC alternative. (b) The use of chaotropic additives (alone or as mixtures) and/or ionic liquids helps in controlling LC retention and peak symmetry, favorably acting on the intrinsic selectivity of the separation; their concentration in the mobile phase will also act as a tool for retention/selectivity control. (c) Both isocratic and gradient elution alternatives may be chosen in the RP separation mode. (d) The use of chaotropism for nicotine RP analysis may be plenarily used with UV detection; when (HR)MS and MS/MS are required as detectors, many degrees of freedom will be lost (with respect to the choice of the chaotropic agent and its concentration in the mobile phase). (e) The HILIC separation alternative offers less flexibility in controlling retention and peak shape. We demonstrated that stationary phases with bimodal (RP/HILIC) working properties can be successfully driven in the HILIC mode to separate nicotine; we also demonstrated that gradient elution and column temperature may control retention and peak symmetry in the HILIC mode. (f) The dilute-and-shoot alternative tested in the RP approach is helpful in analyzing nicotine from e-cigarette liquids; it conserves the ability of selectivity tuning through using chaotropism, provides a basic automation for flow reversal and preserves the stationary phase from deep contamination. Through optimizing the mobile-phase composition profile over the separation period, it is possible to achieve quantitation of many other compounds (with potential analytical importance) existing in the loaded sample. Last, but not least, chaotropic additives in RP mobile phases may represent a valuable alternative for the related impurity assay section in the European Pharmacopoeia (EP) monograph of nicotine [39]. At the moment, the compendial requirements refer to a highly end-capped polar-embedded C18 chemically modified silicagel as a stationary phase exploited with a mobile phase at pH = 10. The harsh pH conditions used in the mobile phase certainly affect the life span of the stationary phase. Stationary phases operated under acidic conditions have extended lifetime periods, and the right choice of chaotropic agents and their concentration tuning in the mobile phase may ensure the required selectivity and adequacy of peak symmetries.

While not the initial aim of this study, the proposed mechanisms should be validated and employed in the analysis of nicotine in e-liquid vapors as well. Furthermore, the analysis can be expanded to analyzing associated impurities and metabolites. Certain impurities, such as anabasine [39,40], have been demonstrated to be teratogenic, and as such, the study of their potential presence in e-liquids can be a major focus for increasing e-cigarette user safety. The methods can potentially be applied to the study of biological samples such as plasma or urine as well.

## Figures and Tables

**Table 1:** Retention and peak shape characteristics for nicotine eluted from the SunFire C18 (150 mm L × 4.6 mm i.d. × 3.5 µm d.p.) chromatographic column, exploited at 25 °C and a flow rate of 1 mL/min. An injection volume of 1 μL of a nicotine 1000 μg/mL solution in methanol was used. Chromatograms were monitored at 260 +/− 4 nm. Potassium nitrate was used as void time indicator.

#	Additive in the Aqueous Component of the Mobile Phase	Organic Modifier	t_R_ (min)	k	S.F.	USP T.F.	N (Plates)
1	H_3_PO_4_ 0.1%	ACN	1.187	0.11	1.120	1.102	3376
2	HClO_4_ 0.1%	ACN	1.774	0.66	1.253	1.198	7622
3	HCOOH 0.1%	ACN	1.128	0.05	0.845	0.929	2362
4	KPF_6_ 20 mM + HClO_4_ 0.1%	ACN	7.216	5.74	1.404	1.212	10,860
5	NH_4_PF_6_ 20 mM + HCOOH 0.1%	ACN	7.792	6.27	1.398	1.210	13,960
6	NH_4_PF_6_ 20 mM + H_3_PO_4_ 0.1%	ACN	6.416	4.99	1.155	1.097	12,259
7	NH_4_BF_4_ 20 mM + HCOOH 0.1%	ACN	2.600	1.43	0.567	0.787	8671
8	NH_4_PF_6_ 20 mM + HCOOH 0.1%	MeOH	4.575	3.27	0.927	0.986	11,405
9	BMPPF_6_ 20 mM + HCOOH 0.1%	MeOH	2.890	1.70	1.027	1.026	10,280

**Table 2:** Calibration data resulting from the experimental design described in the text.

Variable	RP				HILIC			
	Neat Solutions		Toluene Extracts		Neat Solutions		Toluene Extracts	
	Linear	$\mathrm{Weighted}\;(\frac{1}{x^{2}})$	Linear	$\mathrm{Weighted}\;(\frac{1}{x^{2}})$	Linear	$\mathrm{Weighted}\;(\frac{1}{x^{2}})$	Linear	$\mathrm{Weighted}\;(\frac{1}{x^{2}})$
Calibration levels	8	8	8	8	8	8	8	8
x min (μg/mL)	1	1	1	1	1.1	1.1	1	1
x max (μg/mL)	1000	1000	1000	1000	550	550	1000	1000
B (slope)	1.5640	1.5206	2.9334	2.9429	1.3251	1.4527	2.8200	2.6878
A (intercept)	2.4014	−0.5075	1.6295	1.2406	13.6880	2.1176	−0.1448	7.3283
r_xy_ (correlation coeff.)	0.9977	0.9934	0.9999	0.99998	0.9961	0.9948	0.99988	0.9971
S_0_	40.9021	0.1790	1.8391	0.0210	26.0442	0.1537	17.2405	0.2087
S_B_ (st. dev. slope)	0.0430	0.2032	0.0019	0.0238	0.0477	0.1717	0.0181	0.2369
S_A_ (st. dev. intercept)	17.0775	0.5604	0.7679	0.0657	12.4704	0.5231	7.1983	0.6532
LOQ (1)	58.7	1.4	1.6	0.1	53.7	1.4	14.8	1.0
LOQ (2)	109.2	3.7	2.6	0.2	94.1	3.6	25.5	2.4
LOQ (3)	107.7	4.0	2.1	-0.2	83.8	2.1	25.6	−0.3
Bias% min (level)	−188.9 (1)	−15.6 (3)	−13.5 (1)	−1 (4)	−785.2 (1)	−12.3 (4)	259.8 (1)	−13.4 (3)
Bias% max (level)	11.1 (7)	14.7 (7)	0.9 (6)	0.8 (2)	18.7 (6)	14.4 (3)	−2.57 (7)	10.7 (2)
RSD% interval (n = 3)	0.9–4.8 %				0.7–6.8 %			

**Table 3:** E-liquid sample results based on the unweighted linear regressions developed in both RP and HILIC.

E-Cigarette Sample (20 mg/mL)		BI	BR	BT	CT	LB
RP	conc. (μg/mL)	185.91	193.29	187.22	181.99	163.90
	Bias %	−7.0%	−3.4%	−6.4%	−9.0%	−18.1%
HILIC	conc. (μg/mL)	195.20	204.69	199.95	191.09	175.68
	Bias %	−2.4%	2.3%	−0.025%	−4.5%	−12.2%

Blueberry Ice (BI), Berry Twist (BT), Blue Raspberry (BR), Creamy Tobacco (CT), and Lemon Berry (LB).

## Data Availability

The original contributions presented in the study are included in the article/Supplementary Materials; further inquiries can be directed to the corresponding author.

## Supplementary Materials

The following supporting information can be downloaded at https://www.mdpi.com/article/10.3390/molecules30163443/s1, Figure S1: Chromatographic results (isocratic aqueous/organic 95/5 *v*/*v*) for nicotine from a SunFire C18 (150 mm L × 4.6 mm i.d. × 3.5 µm d.p.) chromatographic column, exploited at 25 °C and a flow rate of 1 mL/min. An injection volume of 1 µL of a nicotine 1000 μg/mL solution in methanol was used. Chromatograms were monitored at 260 +/− 4 nm; Figure S2: Chromatographic results produced under isocratic elution of nicotine with a mobile phase containing H_2_O/ACN 95/5 (*v*/*v*) with 0.1% HClO_4_, when alternatively using methanol, acetonitrile and the mobile phase as sample solvent (injected volume 1 µL, nicotine concentration 1 mg/mL). Upper left insert is of the overlaid UV spectra recorded by the diode array detector in the spectral interval ranging from 210 to 500 nm at the times specified by arrows in the chromatograms; Figure S3: Chromatographic results produced under isocratic elution of nicotine with a mobile phase containing H_2_O/ACN 95/5 (*v*/*v*) with 0.1% HCOOH, when alternatively using methanol, acetonitrile and the mobile phase as sample solvent (injected volume 1 µL, nicotine concentration 1 mg/mL); Figure S4: Overlaid chromatograms of nicotine eluted in isocratic conditions (aqueous/organic 95/5 *v*/*v*, 0.1% HCOOH addition, 1 mL/min, 25 °C, 260 nm detection wavelength) when using different concentrations of the chaotropic salt NH_4_PF_6_ in the aqueous component of the mobile phase; Figure S5: Repetitive injections of 1 µL nicotine solution 1 mg/mL dissolved in MeOH, when using 5 mM addition of the chaotropic salt NH_4_PF_6_ in the aqueous component of the mobile phase (aqueous/acetonitrile 0.1% HCOOH: 95/5 *v*/*v*); Figure S6: Chromatograms resulting from injection of different volumes of 1 mg/mL nicotine solutions in different solvents (MeOH, ACN and mobile phase), when using addition of 5 mM of the chaotropic salt NH_4_PF_6_ in the aqueous component of the mobile phase. Arrows in the chromatograms indicate the points at which UV spectra were sampled (overlaid spectra are shown in Figure S7); Figure S7: UV spectra sampled at time points indicated in Figure S6; Figure S8: Isocratic elution of nicotine from a SunFire C18 (150 mm L × 4.6 mm i.d. × 3.5 µm d.p.) chromatographic column, exploited at 25 °C and a flow rate of 1 mL/min. The mobile phase consists of ACN 0.1% HCOOH and aqueous 0.1% HCOOH and 20 mM NH_4_PF_6_ at different volumetric ratios ranging from 2/98 to 22/78; Figure S9: Variation in the retention of nicotine in the SunFire C18 (150 mm L × 4.6 mm i.d. × 3.5 µm d.p.) chromatographic column exploited at temperatures ranging from 10 °C to 50 °C and a flow rate of 1 mL/min. The mobile phase consists of ACN 0.1% HCOOH and aqueous 0.1% HCOOH and 20 mM NH_4_PF_6_ in a volumetric ratio of 5/95; Figure S10: Retention, symmetry and chromatographic efficiency data obtained for nicotine on different SunFire C18 (150 mm L × 4.6 mm i.d. × 3.5 µm d.p.) chromatographic columns (column A—serial no. 01653815614-092; column B—013930085136-73; column C—015233239140-86), exploited at 25 °C and a flow rate of 1 mL/min. The mobile phase consists of ACN 0.1% HCOOH and aqueous 0.1% HCOOH and 20 mM NH_4_PF_6_ in a volumetric ratio of 5/95; Figure S11: Overlaid chromatograms resulting from direct injection of dilute LB liquid (A) and BI-0 liquid (B) with flow reversal after nicotine elution. Elution was kept isocratic at 95/5 aqueous to organic (aq. 0.1% HCOOH and 20 mM NH_4_PF_6_/ACN 0.1% HCOOH) and was followed by a modification to 100% organic modifier after 12 min. Flow rate was 1 mL/min. Upper right inserts illustrate the UV spectra recorded by the diode array detector at the apex of the major peak elution in each chromatogram (retention time interval 0–12 min) in the spectral range 210 to 400 nm; Figure S12: Elution of nicotine (1 µL injection, 1 mg/mL in methanol) under HILIC mode with a mobile phase containing 95% acetonitrile and 5% aqueous 100 mM NH_4_OOCH exploited with 1 mL/min and 30 °C; Figure S13: Elution of nicotine (1 µL injection, 1 mg/mL in toluene) under HILIC mode under two alternative gradient profiles. Profile A: 95% Org. (1.5 min) ↘10%/min↘60% Org. (2 min); 25 °C; Profile B: 98% Org. (2 min) ↘7%/min↘63% Org. (5 min); 50 °C; flow rate: 1 mL/min; Figure S14: Nicotine peak characteristics obtained under HILIC gradient elution profiles (A and B) resulting from the Peak Tools report generated under macro tools.mac running under Chemstation software; Figure S15: Nicotine peak characteristics obtained under HILIC gradient elution profile A on increasingly modifying the column equilibration time periods between successive runs (between 1 and 20 min period); Figure S16: Nicotine peak characteristics obtained under HILIC gradient elution profile B on increasingly modifying the column equilibration time periods between successive runs (between 1 and 20 min period); Figure S17: Nicotine peak characteristics obtained under HILIC gradient elution profile A on increasingly modifying the injected volumes (between 1 and 10 µL). Nicotine solutions were made in toluene; Figure S18: Nicotine peak characteristics obtained under HILIC gradient elution profile A on verifying the instrumental repeatability. Nicotine solutions were made in toluene (1 mg/mL) while the injected volume was 1 µL; Figure S19: Calibration resulting from analysis of neat methanolic solutions of nicotine with concentrations in the 1–1000 µg/mL range under RP separation mechanism; Figure S20: Calibration resulting from analysis of neat methanolic solutions of nicotine with concentrations in the 1–550 µg/mL range under HILIC separation mechanism; Figure S21: Calibration resulting from analysis of extracted toluene solutions of nicotine from the e-cigarette matrix with concentrations in the 1–1000 µg/mL range under RP separation mechanism; Figure S22: Calibration resulting from analysis of extracted toluene solutions of nicotine from the e-cigarette matrix with concentrations in the 1–1000 µg/mL range under HILIC separation mechanism; Figure S23: Possible steric hindrance of the rotation against the sigma bond between the two heterocycles in the structure of nicotine, leading to stabilization of rotameric forms, separable in LC conditions; Table S1: Literature selection about nicotine (alone or together with structurally related compounds) LC analysis, from different matrices, using RP or HILIC separation mechanism and different detection systems; Table S2: Comparison of 25 mM carbonate buffer to sodium hydroxide as diluents, with varying sample/toluene volumes for extraction, with and without saturated sodium chloride; Table S3. Computational formulas used for characterizing the linear regression models used for the experimental calibrations. References citation of [41,42,43,44,45,46,47,48,49,50].

## Abbreviations

The following abbreviations are used in this manuscript:

RP	Reversed Phase
HILIC	Hydrophilic interaction liquid chromatography
LOQ	Limit of quantitation
HRMS	High resolution mass spectrometry
MS-MS	Tandem mass spectrometry
LLE	Liquid–liquid extraction
SPE	Solid-phase extraction
SLE	Supported liquid extraction
EP	European Pharmacopoeia
FID	Flame Ionization Detector
NPD	Nitrogen–Phosphorus Detector (Thermionic Detector)
DAD	Diode array detector
LC	Liquid chromatography
GC	Gas chromatography
AcOEt	Ethyl acetate
MTBE	Methyl tert-butyl ether
BMP	1-methyl-1-butylpyrrolidinium hexafluorophosphate
S.F.	Symmetry factor
USP T	United States Pharmacopeia tailing factor
ODS, C18	Octadecyl-functionalized silicagel
