# Supplementary material for: Reversed-Phase (RP) and Hydrophilic Interaction (HILIC) Separation Mechanisms for the Assay of Nicotine and E-Cigarette Liquids
Source: Molecules. 2025 Aug 21;30(16):3443. doi: 10.3390/molecules30163443 (PMC12388816; doi:10.3390/molecules30163443)
Supplement: Supplementary file 1 [file molecules-30-03443-s001.zip › molecules-3807669-supplementary.pdf]

Supplementary Materials

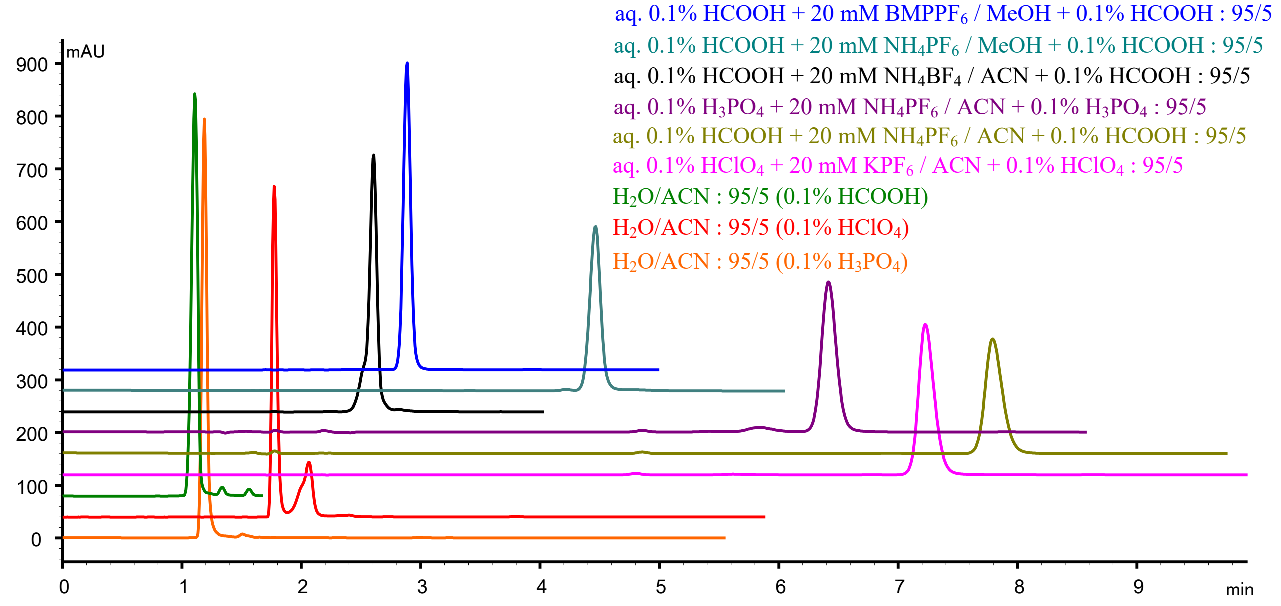

Figure S1. Chromatographic results (isocratic aqueous/organic 95/5 v/v) for nicotine from a SunFire C18 (150 mm L x 4.6 mm i.d. x 3.5 µm d.p.) chromatographic column, exploited at 25 ºC and a flow rate of 1 mL/min. An injection volume of 1 µL of a nicotine 1000 µg/mL solution in methanol was used. Chromatograms were monitored at 260 +/- 4 nm.

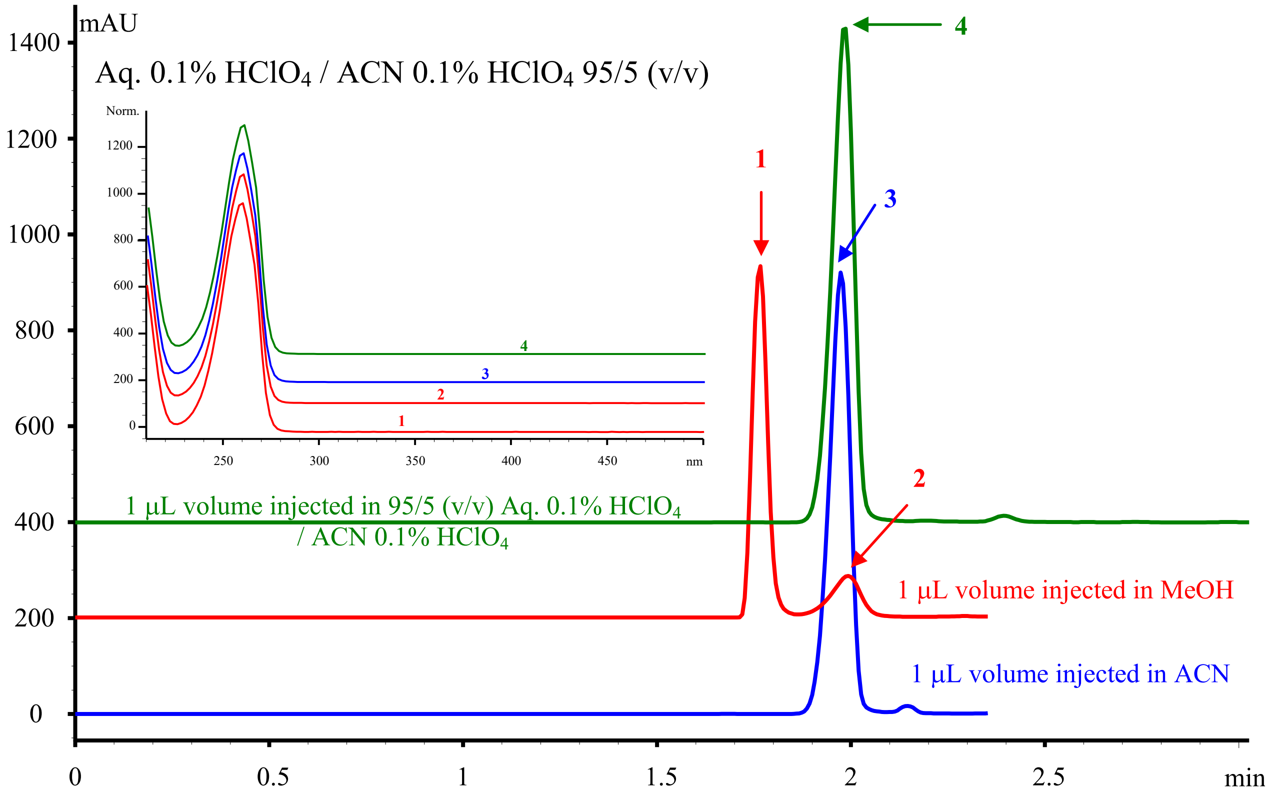

Figure S2. Chromatographic results produced under isocratic elution of nicotine with a mobile phase containing H_2O / ACN 95/5 (v/v) with 0.1% $HClO_4$, when alternatively using methanol, acetonitrile and the mobile phase as sample solvent (injected volume 1 µL, nicotine concentration 1

mg/mL). Upper left insert of the overlaid UV spectra recorded by the Diode Array Detector in the spectral interval ranging from 210 to 500 nm at the times specified by arrows in the chromatograms.

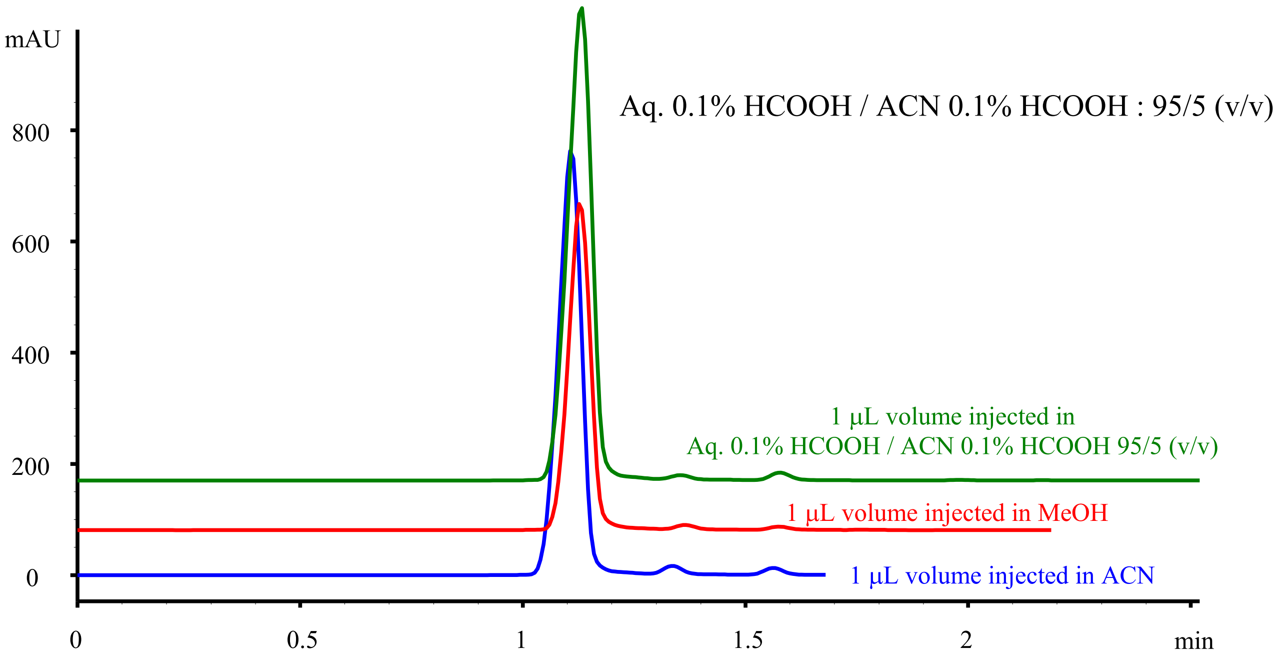

Figure S3. Chromatographic results produced under isocratic elution of nicotine with a mobile phase containing H_2O / ACN 95/5 (v/v) with 0.1% HCOOH, when alternatively using methanol, acetonitrile and the mobile phase as sample solvent (injected volume 1 µL, nicotine concentration 1 mg/mL).

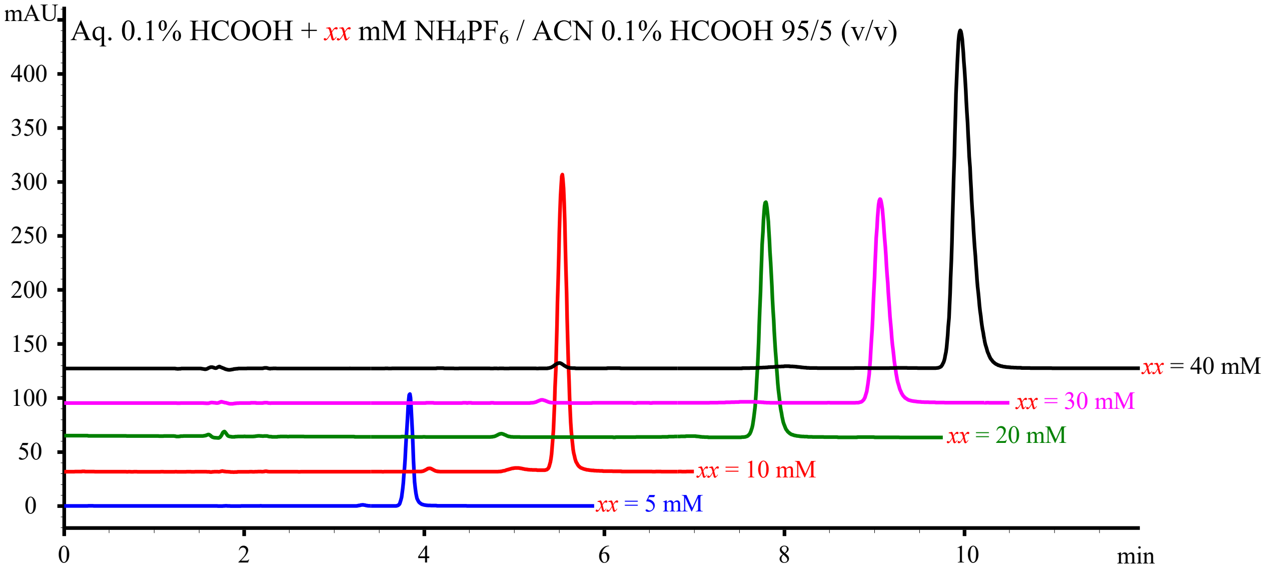

Figure S4. Overlaid chromatograms of nicotine eluted in isocratic conditions (aqueous/organic 95/5 v/v, 0.1% HCOOH addition, 1 mL/min, 25 °C, 260 nm detection wavelength) when using different concentrations of the chaotropic salt NH_4PF_6 in the aqueous component of the mobile phase.

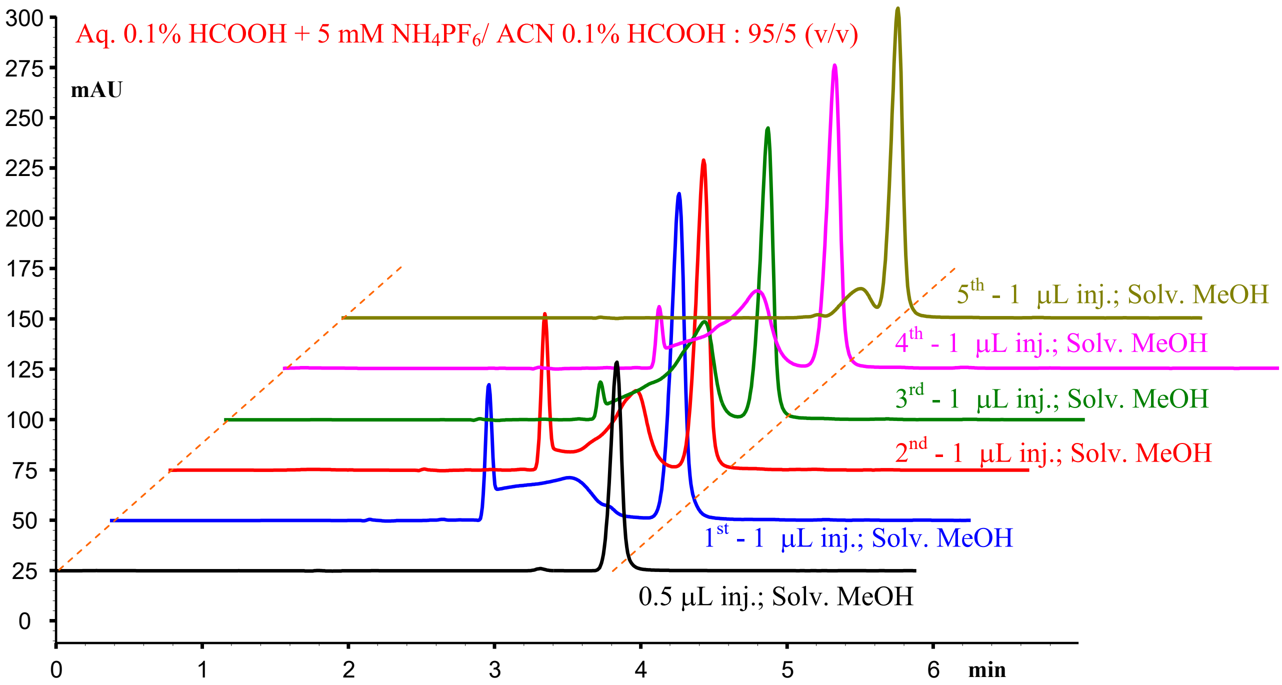

Figure S5. Repetitive injections of 1 µL nicotine solution 1 mg/mL dissolved in MeOH, when using 5 mM addition of the chaotropic salt NH_4PF_6 in the aqueous component of the mobile phase (aqueous/acetonitrile 0.1% HCOOH : 95/5 v/v).

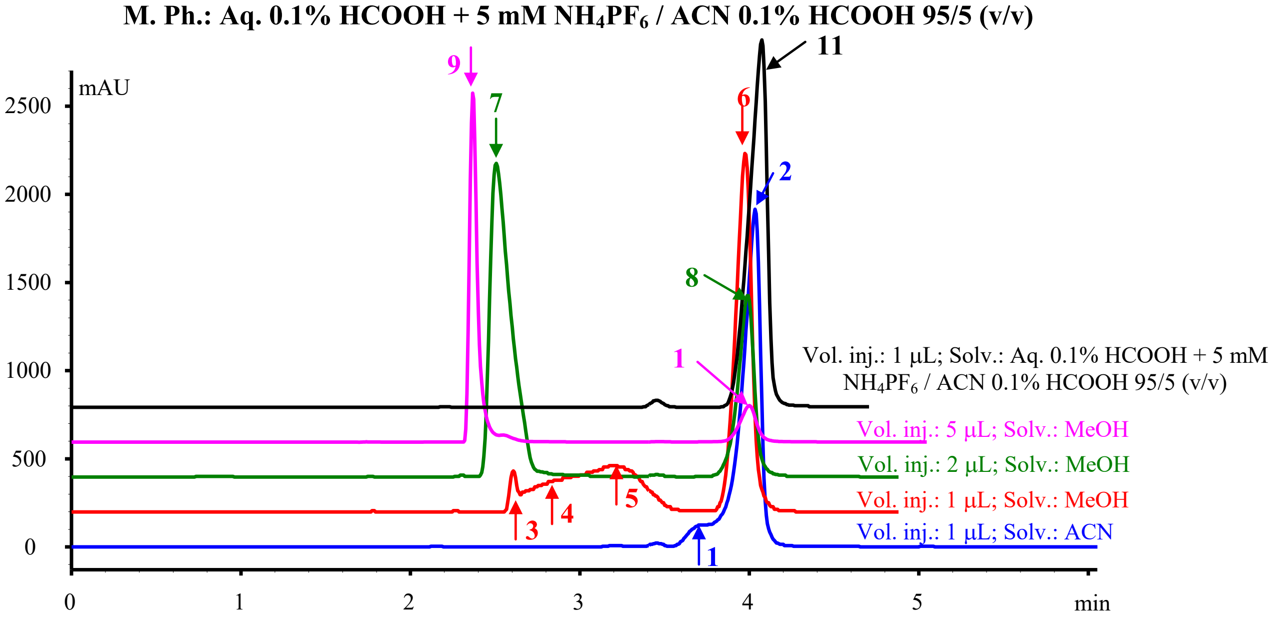

Figure S6. Chromatograms resulting after injection of different volumes of 1 mg/mL nicotine solutions in different solvents (MeOH, ACN and mobile phase), when using addition of 5 mM of the chaotropic salt NH_4PF_6 in the aqueous component of the mobile phase. Arrows in the chromatograms indicate the points in which UV spectra were sampled (overlaid spectra are shown in **Figure S7**).

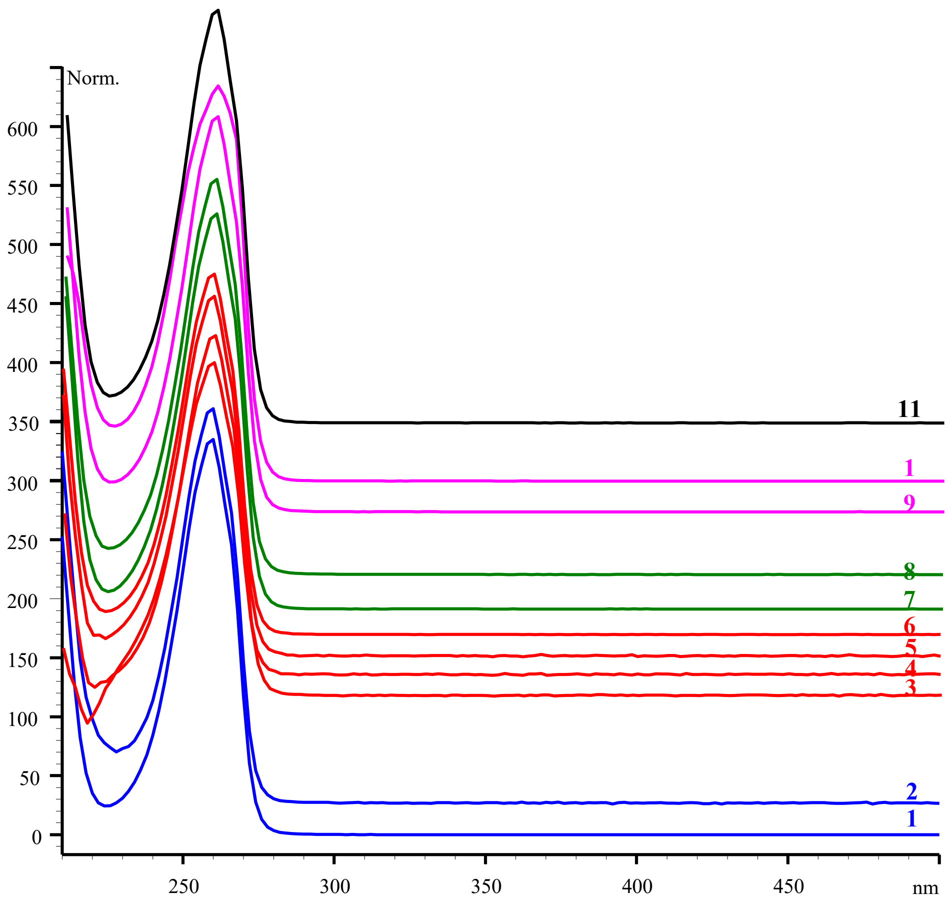

Figure S7. UV spectra sampled at time points indicated in **Figure S6**.

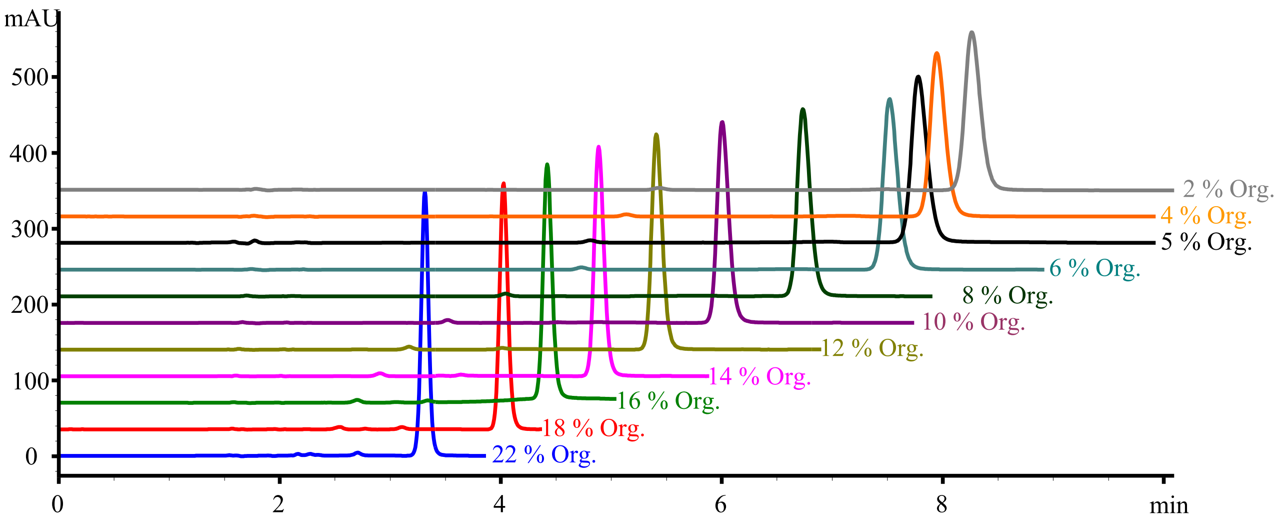

Figure S8. Isocratic elution of nicotine from a SunFire C18 (150 mm L x 4.6 mm i.d. x 3.5 μm d.p.) chromatographic column, exploited at 25 ºC and a flow rate of 1 mL/min. The mobile phase consists in ACN 0.1% HCOOH and aqueous 0.1% HCOOH and 20 mM NH_4PF_6 at different volumetric ratios ranging from 2/98 to 22/78.

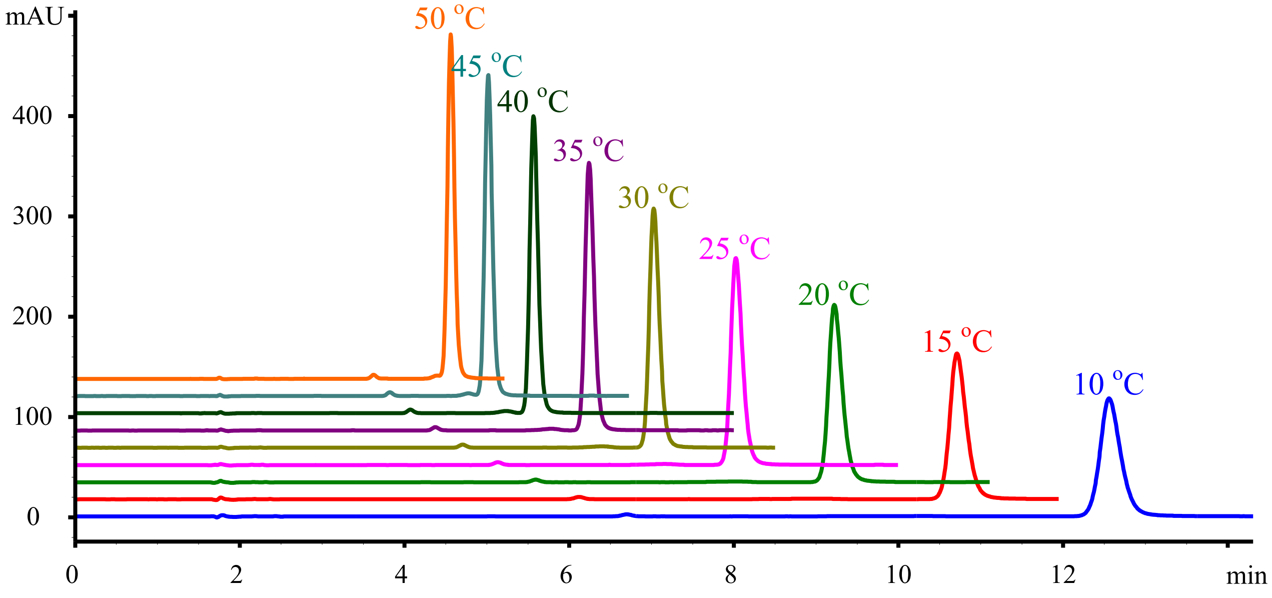

Figure S9. Variation of the retention of nicotine in the SunFire C18 (150 mm L x 4.6 mm i.d. x 3.5 µm d.p.) chromatographic column exploited at temperatures ranging from 10 °C to 50 °C and a flow rate of 1 mL/min. The mobile phase consists in ACN 0.1% HCOOH and aqueous 0.1% HCOOH and 20 mM NH_4PF_6 in a volumetric ratio of 5/95.

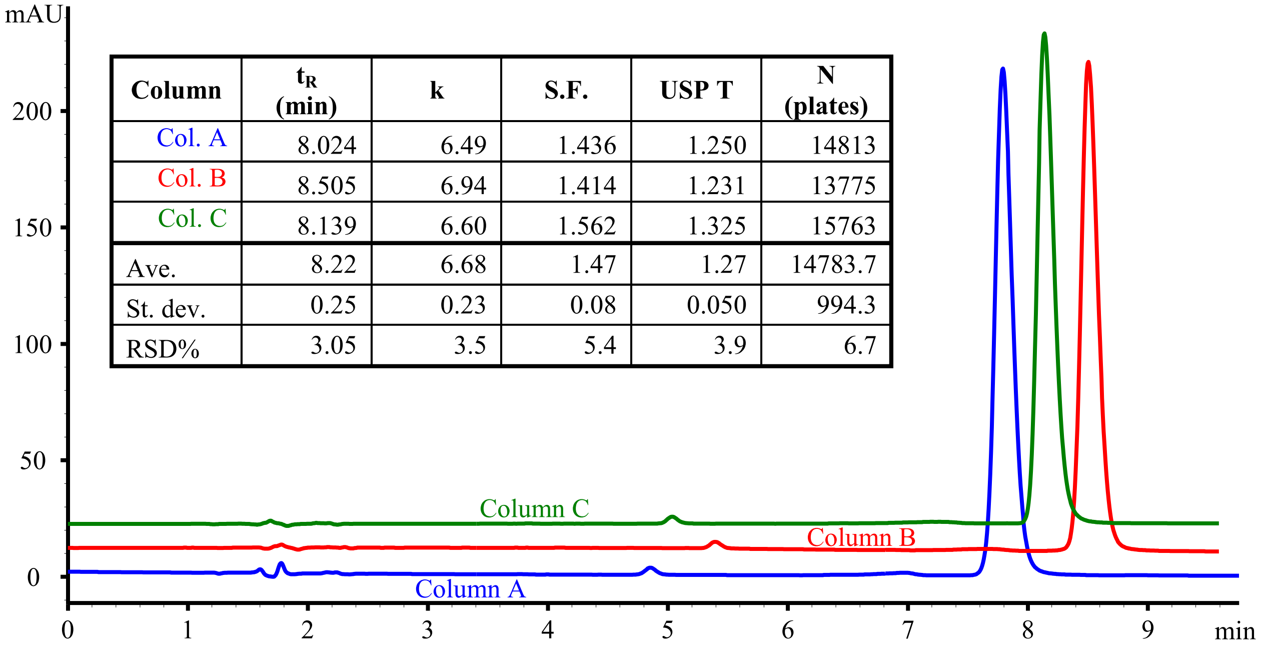

Column	t_R (min)	k	S.F.	USP T	N (plates)
Col. A	8.024	6.49	1.436	1.250	14813
Col. B	8.505	6.94	1.414	1.231	13775
Col. C	8.139	6.60	1.562	1.325	15763
Ave.	8.22	6.68	1.47	1.27	14783.7
St. dev.	0.25	0.23	0.08	0.050	994.3
RSD%	3.05	3.5	5.4	3.9	6.7

Figure S10. Retention, symmetry and chromatographic efficiency data obtained for nicotine on different SunFire C18 (150 mm L x 4.6 mm i.d. x 3.5 µm d.p.) chromatographic columns (column A - serial no. 01653815614-092; column B - 013930085136-73; column C - 015233239140-86), exploited at 25 °C and a flow rate of 1 mL/min. The mobile phase consists of ACN 0.1% HCOOH and aqueous 0.1% HCOOH and 20 mM NH_4PF_6 in a volumetric ratio of 5/95.

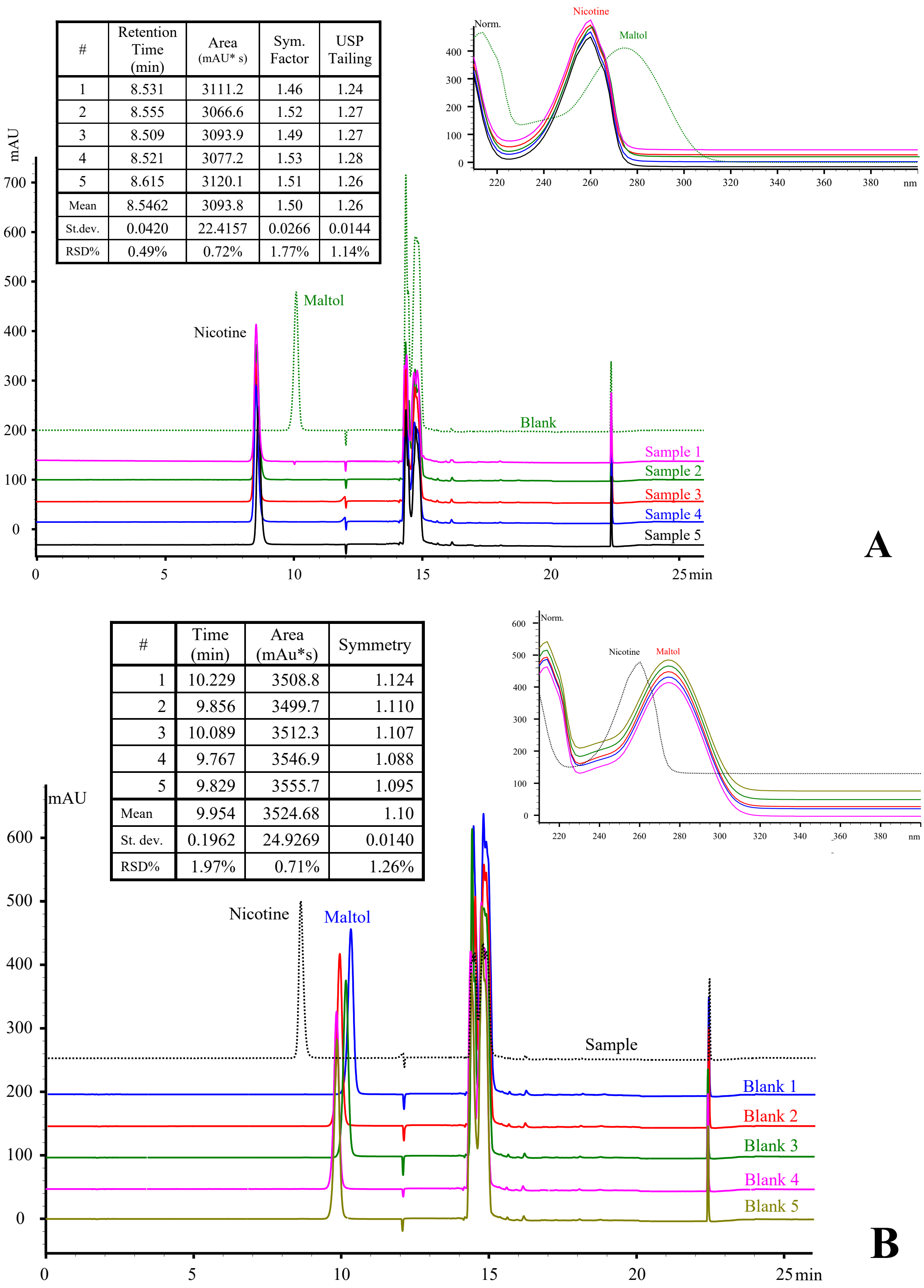

#	Retention Time (min)	Area (mAU* s)	Sym. Factor	USP Tailing
1	8.531	3111.2	1.46	1.24
2	8.555	3066.6	1.52	1.27
3	8.509	3093.9	1.49	1.27
4	8.521	3077.2	1.53	1.28
5	8.615	3120.1	1.51	1.26
Mean	8.5462	3093.8	1.50	1.26
St.dev.	0.0420	22.4157	0.0266	0.0144
RSD%	0.49%	0.72%	1.77%	1.14%

#	Time (min)	Area (mAu*s)	Symmetry
1	10.229	3508.8	1.124
2	9.856	3499.7	1.110
3	10.089	3512.3	1.107
4	9.767	3546.9	1.088
5	9.829	3555.7	1.095
Mean	9.954	3524.68	1.10
St. dev.	0.1962	24.9269	0.0140
RSD%	1.97%	0.71%	1.26%

Figure S11. Overlaid chromatograms resulting after direct injection of dilute LB liquid (A) and BI-0 liquid (B) with flow reversal after nicotine elution. Elution was kept isocratic at 95/5 aqueous to organic (aq. 0.1% HCOOH and 20 mM NH_4PF_6 / ACN 0.1% HCOOH) and was followed by a modification to 100% organic modifier after 12 min. Flow rate was 1 mL/min. Upper right inserts illustrate the UV spectra recorded by the Diode Array Detector at the apex of the major peak eluting in each chromatogram (retention time interval 0-12 min) in the spectral range 210 to 400 nm.

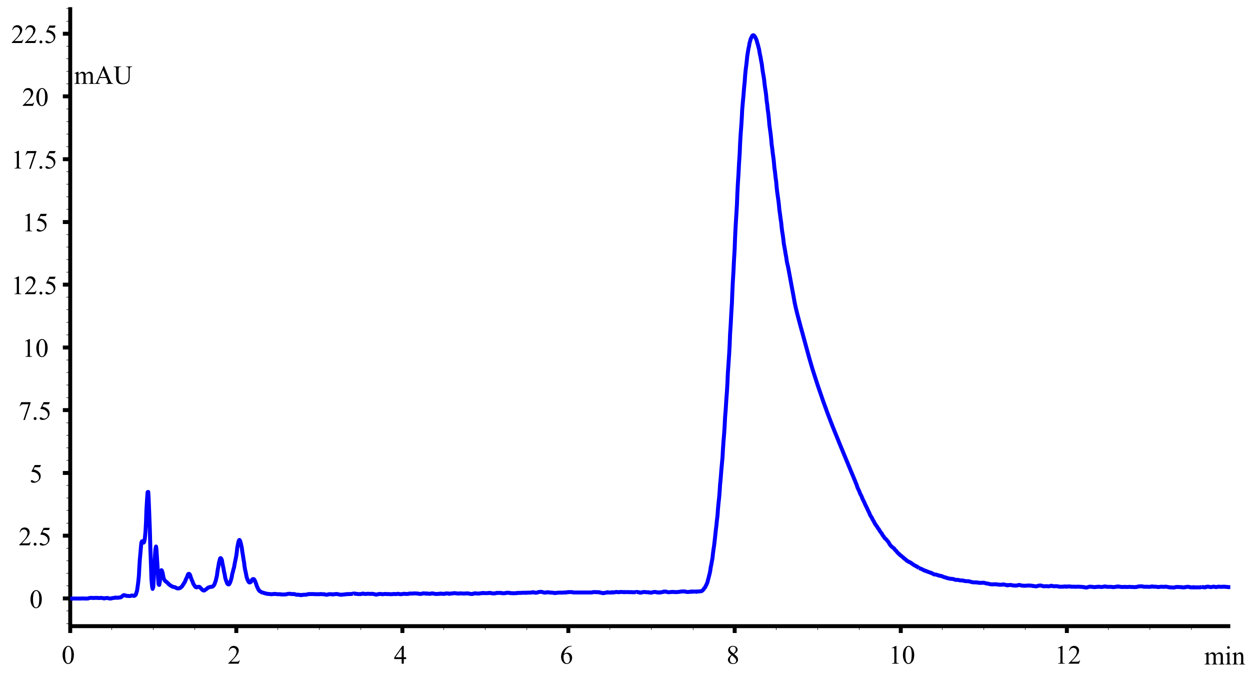

Figure S12. Elution of nicotine (1 µL injection, 1 mg/mL in methanol) under HILIC mode with a mobile phase containing 95% acetonitrile and 5% aqueous 100 mM NH_4OOCH exploited with 1 mL/min and 30 °C.

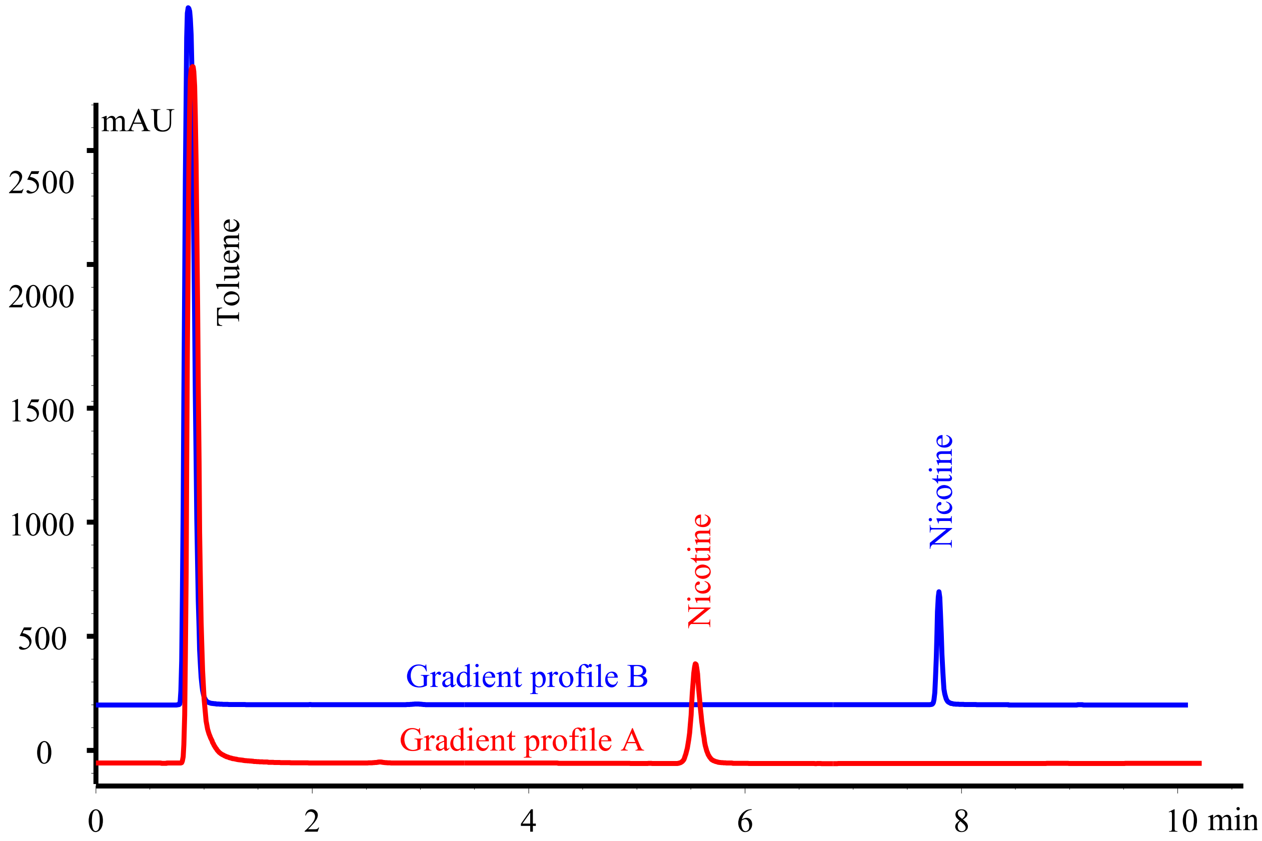

Figure S13. Elution of nicotine (1 µL injection, 1 mg/mL in toluene) under HILIC mode under two alternative gradient profiles. Profile A: 95% Org. (1.5 min) ↘ 10%/min↘ 60% Org. (2 min); 25 °C; Profile B: 98% Org. (2 min) ↘ 7%/min↘ 63% Org. (5 min); 50 °C; Flow rate: 1 mL/min

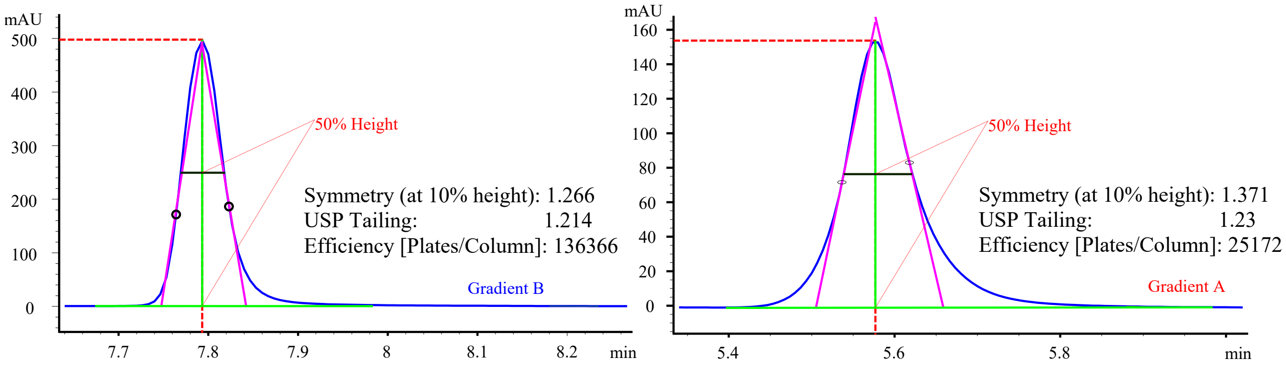

Figure S14. Nicotine peak characteristics obtained under HILIC gradient elution profiles (A and B) as resulting from the Peak Tools report generated under macro tools.mac running under the Chemstation software.

Equil. time (min)	Retention Time (min)	k (apparent)	Area (mAu*s)	Symmetry (at 10% height)	USP Tailing
1	5.583	5.32	945.6	1.423	1.248
2	5.572	5.27	932.7	1.390	1.233
3	5.576	5.29	942.4	1.406	1.235
4	5.578	5.27	942.2	1.394	1.229
5	5.578	5.30	925.9	1.353	1.228
6	5.580	5.20	938.2	1.349	1.226
10	5.580	5.32	934.1	1.376	1.224
20	5.577	5.36	950.8	1.355	1.201
Average	5.578	5.29	939.0	1.381	1.228
St. Dev.	0.003	0.048	7.933	0.027	0.013
RSD%	0.058	0.900	0.845	1.964	1.077

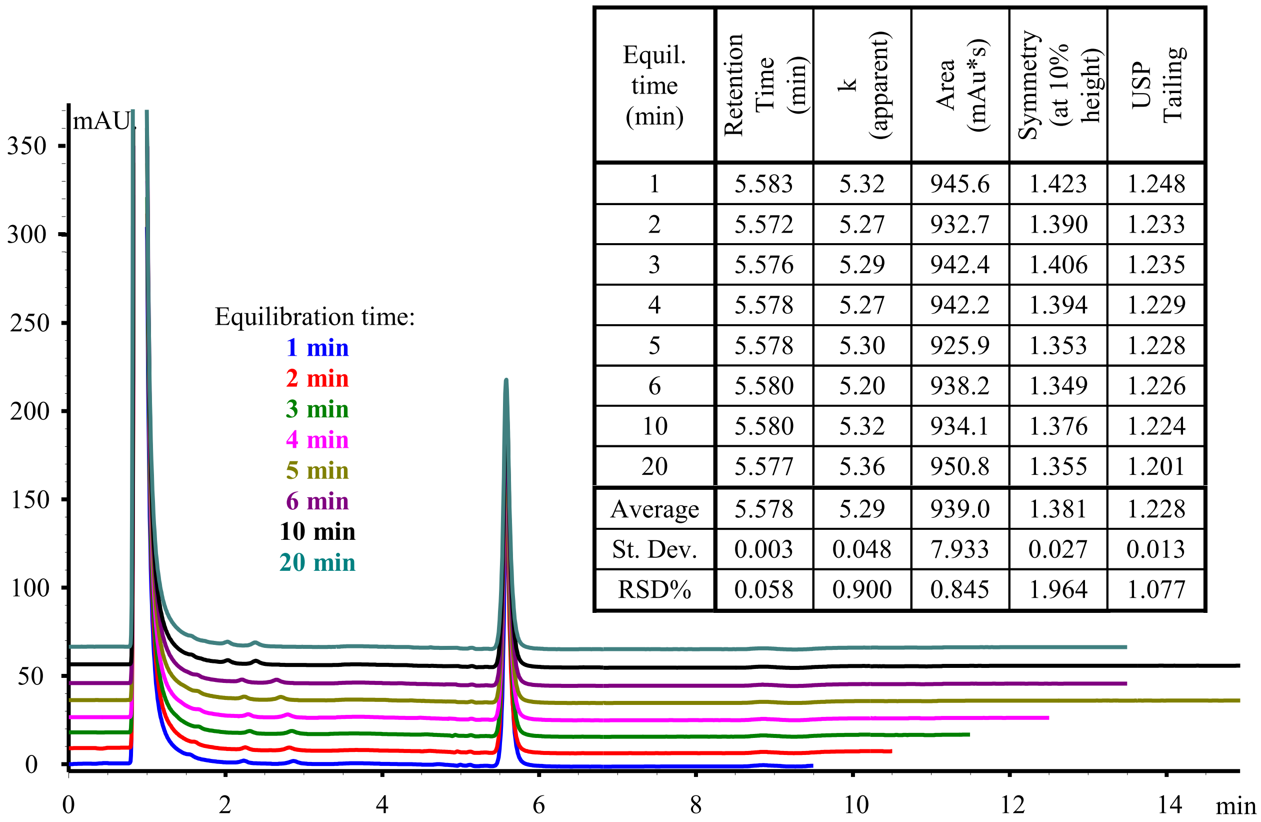

Figure S15. Nicotine peak characteristics obtained under HILIC gradient elution profile A on increasingly modifying the column equilibration time periods between successive runs (between 1- and 20-min period).

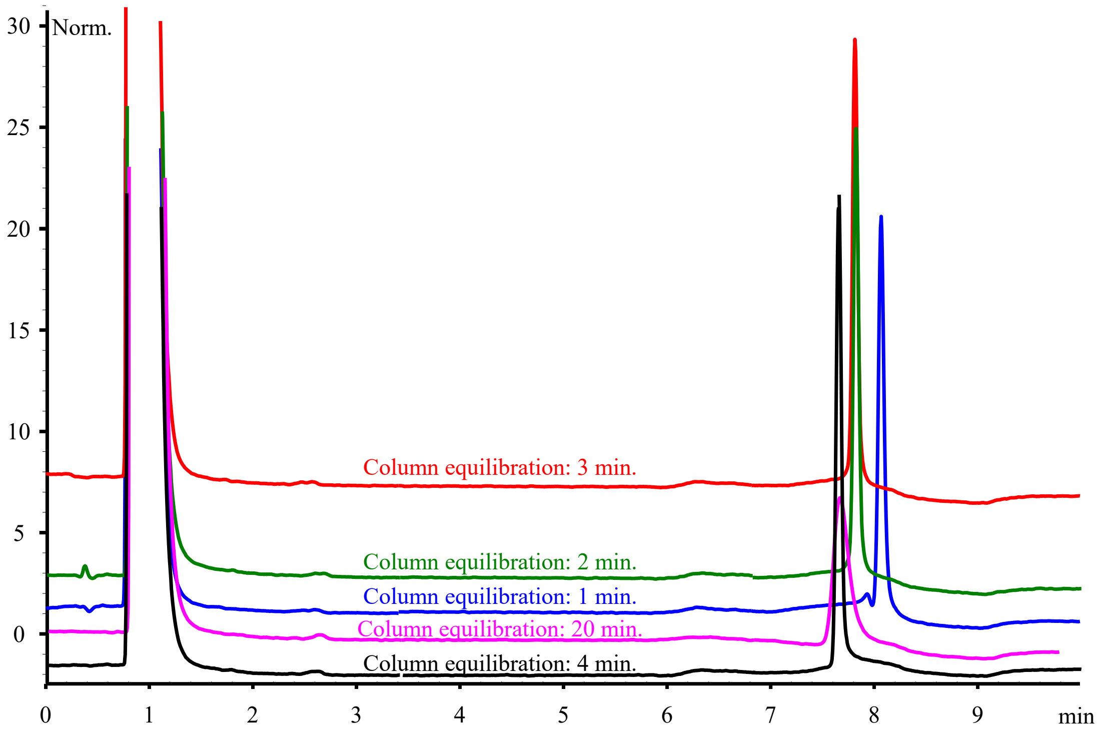

Figure S16. Nicotine peak characteristics obtained under HILIC gradient elution profile B on increasingly modifying the column equilibration time periods between successive runs (between 1- and 20-min period).

Inj. Vol. (µl)	Retention Time t_R (min)	k (apparent)	Symmetry factor 10% height	USP Tailing	N (apparent - plates)
1	5.580	0.839	1.376	1.223	16995
2	5.555	0.842	1.399	1.255	18804
5	5.533	0.826	1.535	1.293	17571
7	5.520	0.829	1.602	1.322	16599
10	5.504	0.821	1.751	1.410	16212
Average	5.538	0.830	1.533	1.301	17236
St. dev.	0.0298	0.0088	0.1540	0.0717	1010
RSD%	0.54	1.06	10.05	5.52	5.86

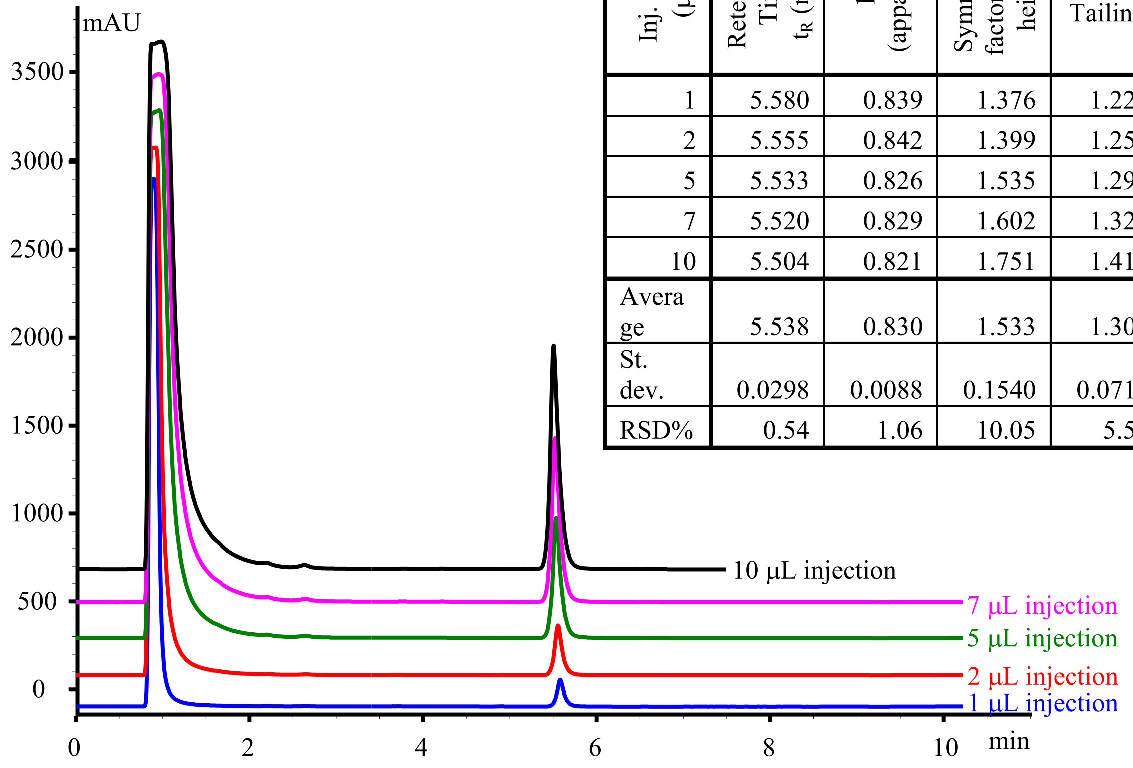

Figure S17. Nicotine peak characteristics obtained under HILIC gradient elution profile A on increasingly modifying the injected volumes (between 1 and 10 µL). Nicotine solutions were made in toluene.

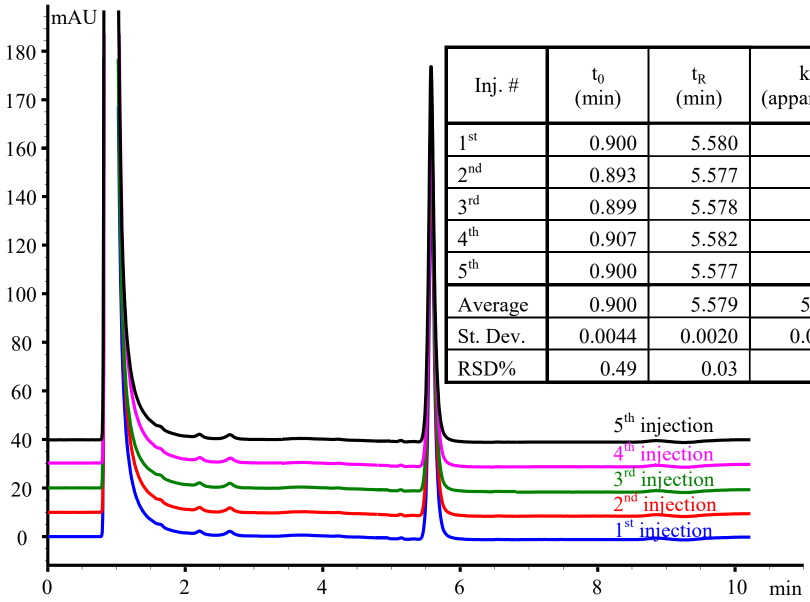

Inj. #	t_0 (min)	t_R (min)	k (apparent)	Peak Area (mAU*s)	Symmetry 10% height	USP Tailing	N apparent (plates)
1st	0.900	5.580	5.20	938.2	1.376	1.223	16995
2nd	0.893	5.577	5.25	972.5	1.454	1.294	16123
3rd	0.899	5.578	5.20	937.7	1.312	1.201	17525
4th	0.907	5.582	5.15	944.7	1.345	1.215	16874
5th	0.900	5.577	5.20	946.9	1.396	1.278	16110
Average	0.900	5.579	5.200	948.000	1.377	1.242	16725.4
St. Dev.	0.0044	0.0020	0.0288	12.7631	0.0480	0.0368	543.3
RSD%	0.49	0.03	0.55	1.35	3.49	2.96	3.25

Figure S18. Nicotine peak characteristics obtained under HILIC gradient elution profile A on verifying the instrumental repeatability. Nicotine solutions were made in toluene (1 mg/mL) while the injected volume was 1 μL.

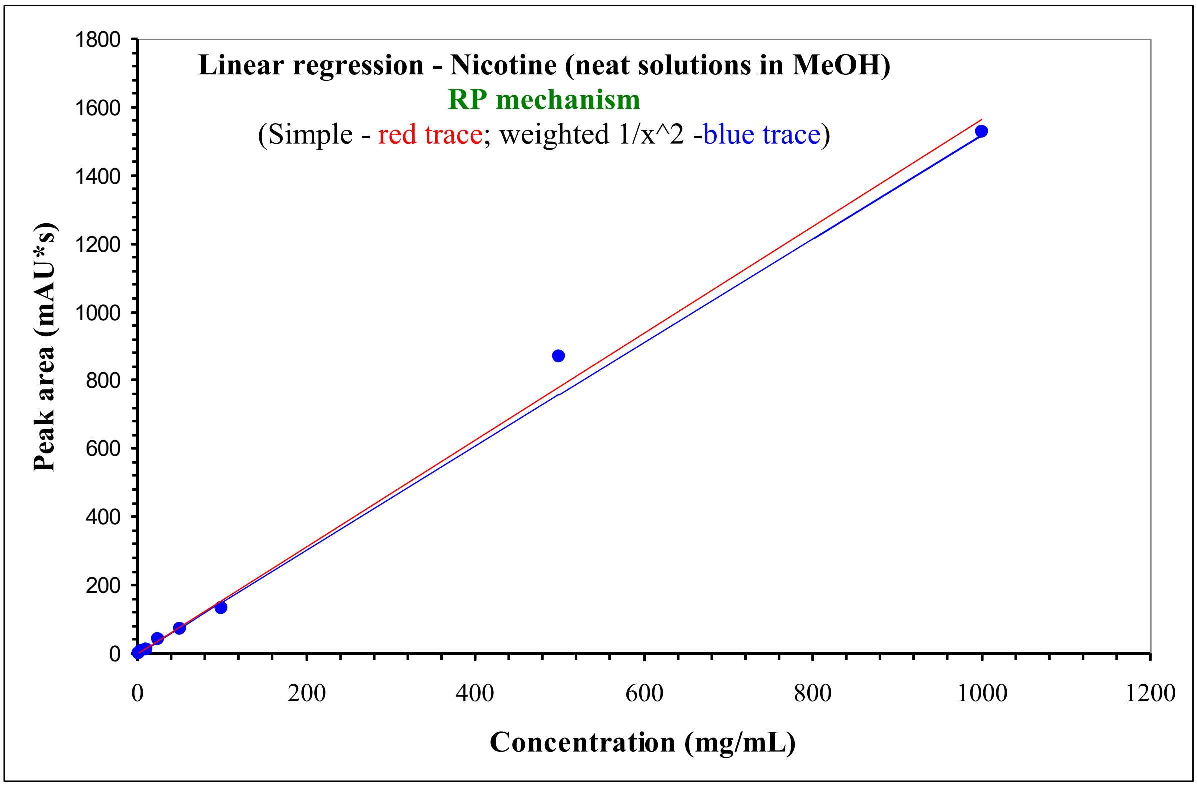

Figure S19. Calibration resulting from analysis of neat methanolic solutions of nicotine with concentrations in the 1-1000 μg/mL range under RP separation mechanism.

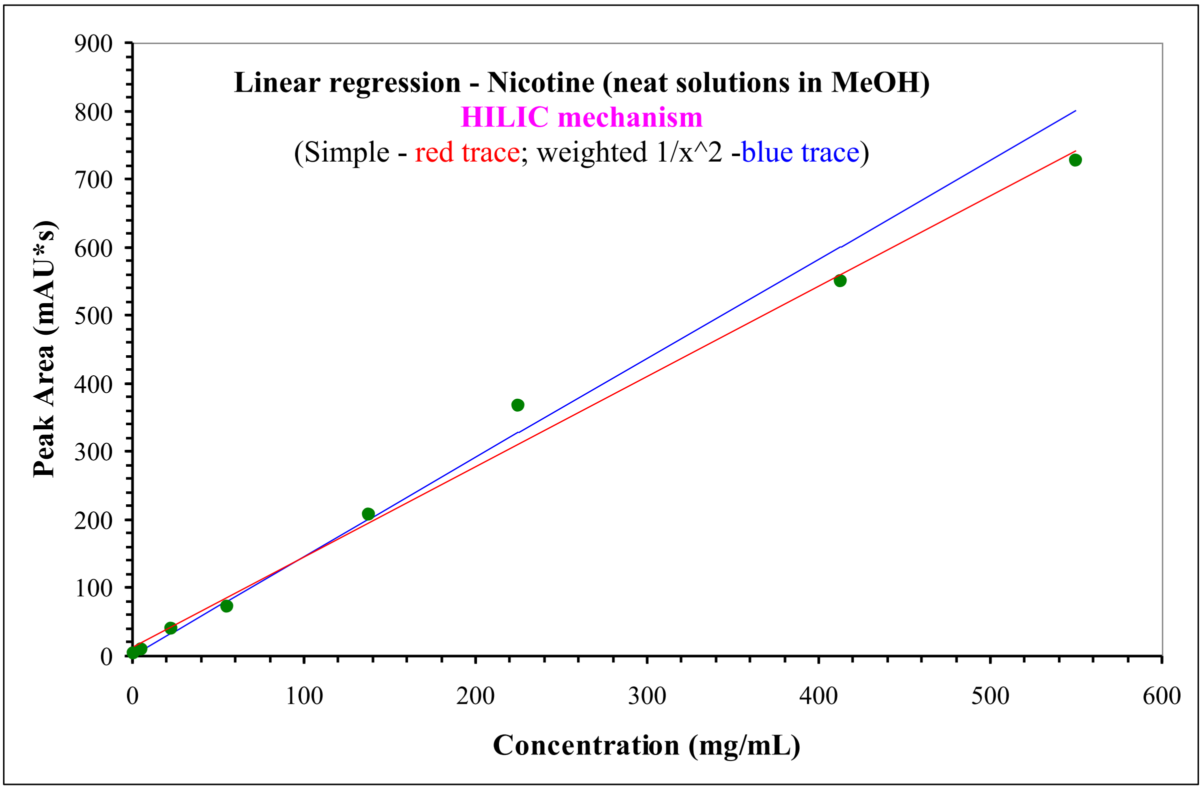

Figure S20. Calibration resulting from analysis of neat methanolic solutions of nicotine with concentrations in the 1-550 µg/mL range under HILIC separation mechanism.

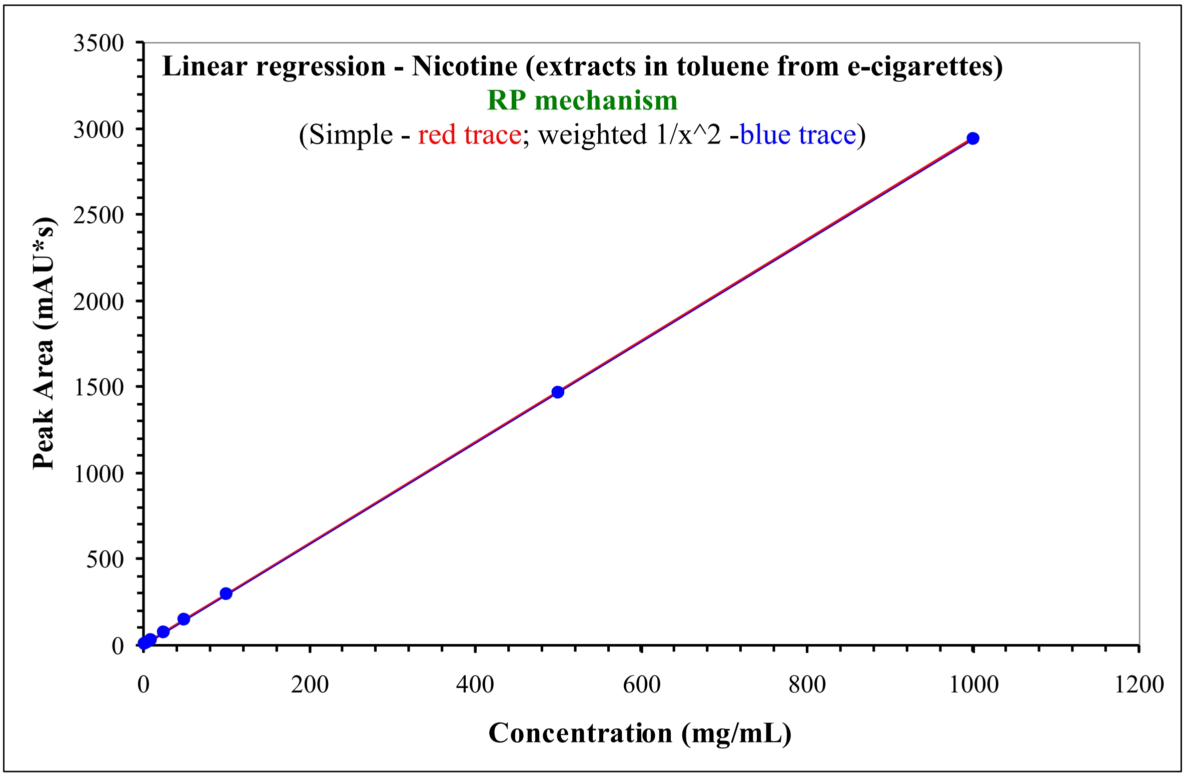

Figure S21. Calibration resulting from analysis of extracted toluene solutions of nicotine from the e-cigarette matrix with concentrations in the 1-1000 µg/mL range under RP separation mechanism.

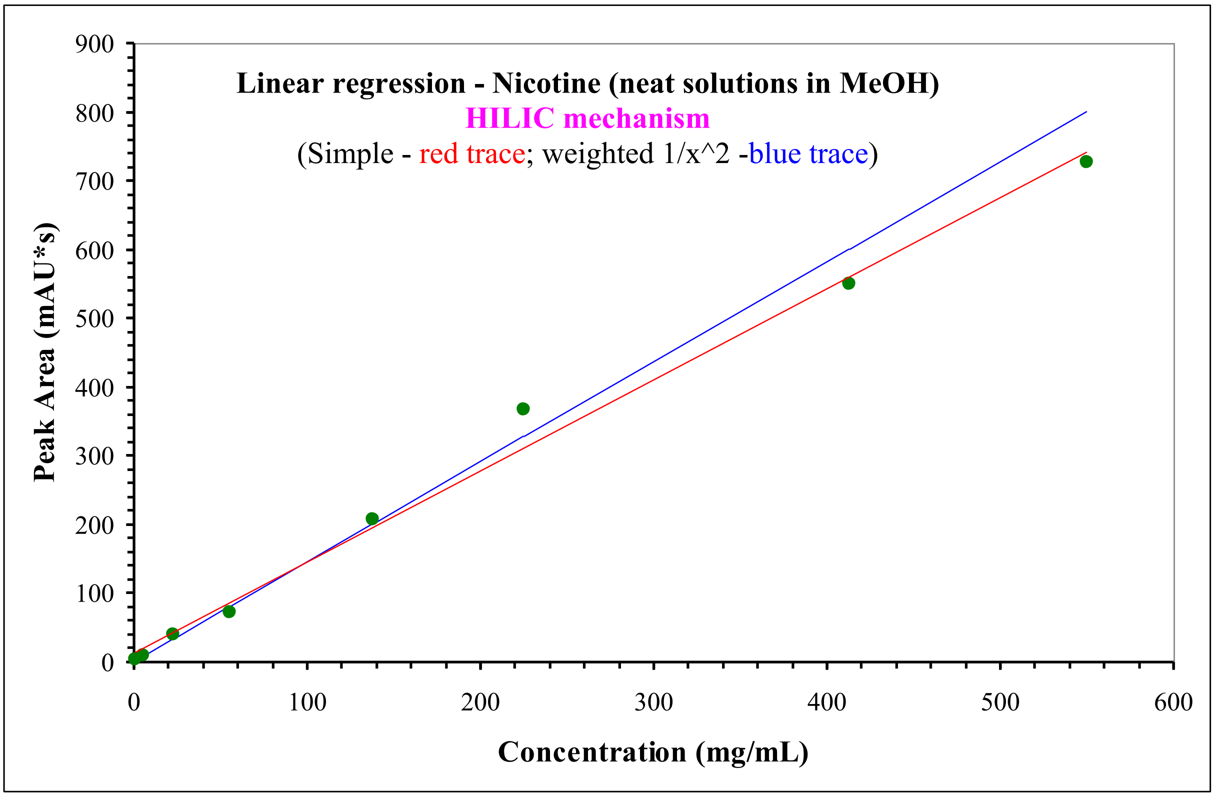

Figure S22. Calibration resulting from analysis of extracted toluene solutions of nicotine from the e-cigarette matrix with concentrations in the 1-1000 µg/mL range under HILIC separation mechanism.

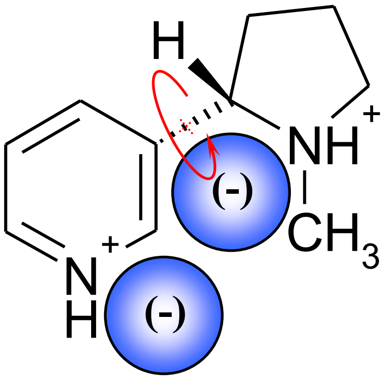

Figure S23. Possible steric hindrance of the rotation against the sigma bond between the two heterocycles in the structure of nicotine, leading to stabilization of rotameric forms, separable in LC conditions.

Table S1. Literature selection about nicotine (alone or together with structural related compounds) LC analysis, from different matrices, using RP or HILIC separation mechanism and different detection systems.

#	Analyte(s)	Matrix	LC separation mechanism	Column	Mobile Phase composition	Elution mode	Detection	Ref.
1.	Nicotine and cotinine	Human plasma	RP	LiChrospher® RP-18 Column	Aqueous 0.5 / 1 / 2.5 mM of 1-butyl-3- methyl imidazolium hexafluoro phosphate or tetrafluoroborate (pH = 2.3 or 2.5 with phosphoric acid or phosphate buffer) and acetonitrile	Isocratic	UV 260 nm	28
2.	Nicotine, cotinine, trans-3'-hydroxy cotinine	Human plasma	RP	Agilent 5 HC–C18(2)<br><br>ZORBAX RRHD StableBond C18	Aqueous 20 mM phosphate buffer (pH = 2.7) and 30 mM sodium hexafluorophosphate<br>Aqueous 5 mM ammonium formate pH = 4.5 and acetonitrile	Isocratic<br><br>Isocratic	UV 260 nm<br><br>ESI/MS-MS	29
3.	Nicotine, cotinine, trans-3'-hydroxy cotinine	Human plasma	RP<br><br>HILIC	Luna Phenyl-Hexyl<br>EC Nucleosil 100–5 HD C18<br>EC Nucleosil 120–3 C8<br>Luna HILIC	Aqueous 5 mM Ammonium acetate at variable pH values (3 to 4.6) and acetonitrile or methanol	Gradient<br><br>Isocratic	ESI/MS-MS	33
4.	Nicotine and related impurities	APIs and formulations	RP	End capped polar embeded C18	Aqueous ammonium acetate pH = 10 and acetonitrile	gradient	UV 254 nm	39
5.	Nicotine	Zero nicotine e-cigatte liquids	RP<br><br>HILIC	Zorbax XDB C18<br><br>Ascentis Si	Aqueous 0.05% formic acid and 0.1% formic acid in methanol<br>Aqueous 10 mM ammonium formate and acetonitrile	Isocratic<br><br>Isocratic	ESI/MS-MS	41
6.	Nicotine, cotinine, and 3′-hydroxycotinine	Serum, saliva	RP	XBridge BEH C18 with a XBridge BEH C18 3.5 µm guard column	Aqueous 0.01% ammonium hydroxide (pH = 10.5) and 0.01% ammonium hydroxide in methanol	Gradient	ESI/MS-MS	42
7.	Nicotine and its main	Human serum	HILIC	Luna HILIC	Aqueous 100 mM ammonium	Isocratic	ESI/MS-MS	43

	metabolites				formate and acetonitrile			
8.	Nicotine, cotinine, and tobacco-specific carcinogens	Serum	HILIC	Acquity UPLC BEH HILIC	Aqueous 10 mM ammonium formate (pH = 3) and 0.1% formic acid	Gradient	ESI/MS-MS	44
9.	Nicotine, cotinine, trans-3'-hydroxy cotinine and varenicline	Human plasma	HILIC	HILIC BEH	Aqueous ammonium formate pH = 3 and acetonitrile	Gradient	ESI/MS-MS	45
10.	Nicotine, cotinine and different other APIs	Bulk	HILIC	Betasil silica	Acetonitrile / Aqueous 0.025 or 0.05% trifluoroacetic acid, 0.5% acetic acid and 1% propionic acid	Isocratic	ESI/MS-MS	46
11	Nicotine, cotinine, trans-3-hydroxycotinine, nornicotine, anatabine, anabasine, amino ketone (4-(methyl amino)-1-(3-pyridyl) 1-butanone), 2-hydroxynicotine and 6-hydroxynicotine	Tobacco leaves	RP	Waters Xbridge Amide coupled with Atlantis T3 (C18)	Aqueous 100 mM ammonium formate pH = 4.5 and acetonitrile	Gradient	ESI/MS-MS	47
12	Nicotine and 10 related metabolites	Urine	RP	Luna C8	Aqueous 0.1% ammonium acetate (pH = 7) and methanol	Gradient	ESI/MS-MS	48
13	Nicotine and its major metabolites	Whole blood	RP	Accucore Phenyl Hexyl	Aqueous 5 mM ammonium acetate buffer pH = 3.5 and ACN	Gradient	ESI/(HR)MS-MS	49
14	Nicotine and cotinine	Human serum	RP	X-Bridge (Waters) C18	Acetonitrile	Isocratic	MS	50

Table S2. Comparison of 25 mM carbonate buffer to sodium hydroxide as diluents, with varying sample:toluene volumes for extraction, with and without saturated sodium chloride.

**25 mM Carbonate ($NaHCO_3$/Na_2CO_3) buffer**				
	**Neat (C)**		**With Salt (CS)**	
(v/v)	Area	RSD %	Area	RSD %
1/1	762.667	1.54%	535.025	13.87%
2/1	1429.8	7.09%	1661.85	47.03%
4/1	2892.05	28.32%	2043.225	20.76%
**Sodium Hydroxide**				
	**Neat (H)**		**With Salt (HS)**	
(v/v)	Area	RSD %	Area	RSD %
1/1	451.3	1.54%	373.175	32.90%
2/1	833.175	1.57%	896.025	4.73%
4/1	1271.4	9.43%	1747.175	23.47%

Table S3. Computational formulas used for characterizing the linear regression models used for the experimental calibrations.

Characteristic	Notation	Computational relationships Linear regression model	Computational relationships Weighted linear regression model
Standard deviation of the independent variable population	S_x	$s_x = \sqrt{\frac{\sum_{i=1}^{n} x_i^2 - \frac{\left(\sum_{i=1}^{n} x_i\right)^2}{n}}{n-1}}$	$S_x = \sqrt{\frac{\sum(x^2 \times w) - \frac{(\sum x \times w)^2}{w}}{n-1}}$
Standard deviation of the dependent variable population	S_y	$s_y = \sqrt{\frac{\sum_{i=1}^{n} y_i^2 - \frac{\left(\sum_{i=1}^{n} y_i\right)^2}{n}}{n-1}}$	$S_y = \sqrt{\frac{\sum(y^2 \times w) - \frac{(\sum y \times w)^2}{w}}{n-1}}$
Covariance	S_{xy}	$s_{xy} = \frac{\sum_{i=1}^{n} x_i \times y_i - \frac{\sum_{i=1}^{n} x_i \times \sum_{i=1}^{n} y_i}{n}}{n-1}$	$S_{xy} = \frac{\sum x \times y \times w - \frac{(\sum x \times w) \times (\sum y \times w)}{\sum w}}{n-1}$
Correlation coefficient	r_{xy}	$r_{xy} = \frac{s_{xy}}{s_x \times s_y}$	$r_{xy} = \frac{s_{xy}}{s_x \times s_y}$
Slope	B	$B = \frac{\sum_{i=1}^{n} x_i \times y_i - \frac{\sum_{i=1}^{n} x_i \times \sum_{i=1}^{n} y_i}{n}}{\sum_{i=1}^{n} x_i^2 - \frac{\left(\sum_{i=1}^{n} x_i\right)^2}{n}}$	$B = \frac{\sum(x \times y \times w) - \frac{(\sum(x \times w)) \times (\sum(y \times w))}{\sum w}}{\sum(x^2 \times w) - \frac{(\sum x \times w)^2}{\sum w}}$
Intercept	A	$A = \frac{\sum_{i=1}^{n} y_i}{n} - B \times \frac{\sum_{i=1}^{n} x_i}{n}$	$A = \frac{\sum y \times w}{\sum w} - B \times \frac{\sum x \times w}{\sum w}$
Standard deviation of the whole y values population	S_0	$s_0^2 = \frac{\sum_{i=1}^{n} y_i^2 - A \times \sum_{i=1}^{n} y_i - B \times \sum_{i=1}^{n} x_i \times y_i}{n-2}$	$S_0^2 = \frac{\left(\sum(y^2 \times w) - \frac{(\sum y \times w)^2}{\sum w}\right) - B \times \left(\sum(x \times y \times w) - \frac{(\sum x \times w) \times (\sum y \times w)}{\sum w}\right)}{n-2}$
Standard deviation of the slope	S_B	$s_B^2 = \frac{n \times s_0^2}{n \times \sum_{i=1}^{n} x_i^2 - \left(\sum_{i=1}^{n} x_i\right)^2}$	$S_B^2 = \frac{(\sum w) \times S_0^2}{(\sum w) \times \sum(x^2 \times w) - (\sum x \times w)^2}$
Standard deviation of the intercept	S_A	$s_A^2 = \frac{s_B^2}{n} \times \sum_{i=1}^{n} x_i^2$	$S_A^2 = S_B^2 \times \frac{\sum(x^2 \times w)}{\sum w}$
Limit of quantitation (version 1)	LOQ(1)	$LOQ = \frac{2 \times t \times \left(s_A + \frac{\sum_{i=1}^{n} x_i \times s_B}{n}\right)}{B + 2 \times t \times s_B}$	$LOQ = \frac{2 \times t \times \left(S_A + \frac{\sum x \times w}{\sum w} \times S_B\right)}{B + 2 \times t \times S_B}$
Limit of quantitation (version 2)	LOQ(2)	$LOQ = \frac{10 \times s_A}{B}$	$LOQ = \frac{10 \times s_A}{B}$
Limit of quantitation (version 3)	LOQ(3)	$LOQ = \frac{10 \times s_A - A}{B}$	$LOQ = \frac{10 \times s_A - A}{B}$
Back-interpolated concentration value	$C_{b.i.}$	$c_{b.i.} = \frac{y_i - A}{B}$	$c_{b.i.} = \frac{y_i - A}{B}$
Accuracy	% bias	$\%bias = \frac{c_{b.i.} - x_i}{x_i} \times 100$	$\%bias = \frac{c_{b.i.} - x_i}{x_i} \times 100$
